# Association between air pollution and primary liver cancer in European and east Asian populations: a Mendelian randomization study

**DOI:** 10.3389/fpubh.2023.1212301

**Published:** 2023-07-27

**Authors:** Mengting Sun, Ming Gao, Manjun Luo, Tingting Wang, Taowei Zhong, Jiabi Qin

**Affiliations:** ^1^Department of Epidemiology and Health Statistics, Xiangya School of Public Health, Central South University, Changsha, China; ^2^Department of Geriatric Medicine, Center of Coronary Circulation, Xiangya Hospital, Central South University, Changsha, Hunan, China; ^3^National Health Committee (NHC) Key Laboratory of Birth Defect for Research and Prevention, Hunan Provincial Maternal and Child Health Care Hospital, Changsha, Hunan, China; ^4^Hunan Provincial Key Laboratory of Clinical Epidemiology, Changsha, Hunan, China

**Keywords:** air pollution, primary liver cancer, particulate matter, nitrogen oxides, biomarkers

## Abstract

**Purpose:**

The incidence of primary liver cancer is increasing year by year, with environmental factors playing a non-negligible role. At present, many studies are still disputing whether air pollution is associated with primary liver cancer incidence, and it is difficult to draw causal inferences. Therefore, in this study, we used two-sample Mendelian randomization (MR) to assess the causal relationship between air pollution (including PM2.5, PM2.5–10, PM10, nitrogen dioxide and nitrogen oxides) and primary liver cancer risk and its related biomarkers (Alpha-fetoprotein, Osteopontin, Glypican-3 and Arginase-1).

**Patients and methods:**

We used large-scale publicly available genome-wide association studies (GWAS) summary data to conduct MR analyses of European and East Asian populations. Inverse variance weighted (IVW) method was used as the main analysis method, and weighted median model, MR-Egger, simple model and weighted model methods were selected for quality control. Heterogeneity was checked by the Cochran’s Q test. The MR-Egger regression and the MR-PRESSO global test detect pleiotropy. The sensitivity analysis was performed using the leave-one-out method.

**Results:**

Between air pollution and primary liver cancer in either European (PM2.5: *p* = 0.993; PM2.5–10: *p* = 0.833; PM10: *p* = 0.257; nitrogen dioxide: *p* = 0.215; nitrogen oxides: *p* = 0.614) or East Asian (PM2.5: *p* = 0.718; PM2.5–10: *p* = 0.362; PM10: *p* = 0.720; nitrogen dioxide: *p* = 0.101; nitrogen oxides: *p* = 0.760) populations were found no statistical association. Notably, there was a causal relationship between nitrogen oxides and Arginase-1, a biomarker associated with hepatocellular differentiation, statistically significant associations remained after deletion for single nucleotide polymorphisms (SNPs) associated with alcohol intake frequency, Body mass index (BMI) and cancers (Beta: 4.46; 95%CI: 0.83–8.08; *p* = 0.015). There was no heterogeneity or pleiotropy in the results.

**Conclusion:**

This MR study found no evidence to support a causality between air pollution and primary liver cancer in European and East Asian populations, but nitrogen oxides may affect hepatocellular differentiation.

## 1. Introduction

Primary liver cancer is a malignant tumor originating from liver cells or intrahepatic bile duct epithelial cells, which is composed of hepatocellular carcinoma, cholangiocarcinoma, and mixed carcinoma (1). The estimated global cancer incidence rate for primary liver cancer in 2018 was 9.3 per 100,000 person-years, with a corresponding mortality rate of 8.5, making it the sixth most common cancer and the fourth leading cause of cancer death globally (2, 3). Currently, the region with the highest rates and prevalence of primary liver cancer is East Asia. However, from 1978 to 2012, the incidence of primary liver cancer has also increased year-on-year in regions with low incidence rates, such as most European countries, India and the United States, placing a huge burden on individuals, families and society (4). Diagnosis of primary liver cancer relies on pathological biopsies, and the detection of tumor biomarkers also plays an important role in early screening, diagnosis, treatment assessment, recurrence and prognosis prediction of tumors (1). Alpha-fetoprotein (AFP), Osteopontin (OPN), Glypican-3 (GPC-3) and Arginase-1 (Arg-1) are common primary liver cancer related biomarkers. AFP, derived from fetal hepatocytes and yolk sacs, has been widely used as a useful cancer biomarker in the diagnosis of liver cancers (5). OPN is a highly modified, phosphorylated and glycosylated extracellular matrix protein that binds to integrins and is expressed in a variety of cells. When combined with AFP, the sensitivity of OPN in the diagnosis of liver cancers increases to 65% (6). GPC-3, a membrane-bound heparin sulfate proteoglycan belonging to the glycoprotein family, is over-expressed in up to 80% of patients with hepatocellular carcinoma and can distinguish liver cancer from other malignancies (7). Arg-1, an enzyme associated with the hydrolysis of arginine to ornithine and urea, is highly sensitive and specific for the detection of malignant hepatocytes and is considered a useful biomarker of hepatocellular differentiation (8, 9). Hepatitis B virus (HBV) infection is the most important driver of primary liver cancer, but with the increase of HBV vaccination coverage, HBV infection has been effectively controlled, and the increase in incidence and mortality from primary liver cancer may be more attributable to other factors such as smoking, alcohol consumption and dietary habits (10, 11). In addition, the impact of environmental factors cannot be ignored as industrialization and associated pollution from burning fossil fuels, or coal, oil and gas, and vehicle emissions increase.

As a long-standing and widespread industrial pollutant, air pollution poses a worrying health hazard. Air pollutants are usually classified as particulate matter (PM), or as gases such as nitrogen dioxide (NO_2_), nitrogen oxides (NO_x_), sulfur dioxide (SO_2_), carbon monoxide (CO), and ozone (O_3_), etc. (12). PM2.5 is the most frequently inspected pollutant, followed by NO_2_ and NO_x_, with few studies focusing on other pollutants. From 1990 to 2015, the death rate attributable to PM2.5 increased from 3.5 to 4.2 million, accounting for 7.6 percent of the total global deaths (13). The high health hazard of PM2.5 is the main reason for its widespread concern. The International Agency for Research on Cancer (IARC) classifies air pollutants as Group I human carcinogens, and numerous studies have shown a positive association between air pollution and some cancers, such as lung, kidney and breast cancer (14–16).

There is no consensus on the association between air pollution and the risk of primary liver cancer. Although a study involving 20,221 participants in the United States found a positive association between PM2.5 and liver cancer mortality (HR was 1.18 by 5 μg/m^3^ increase in PM2.5; 95% CI: 1.16–1.20) (17), among the 8 studies on PM2.5 and the risk of primary liver cancer, only three studies (Coleman et al., Pan et al. and Vo Pham et al.) showed significant association between PM2.5 and primary liver cancer risk, while the other five studies did not (18–22). However, in five studies on the association between NO_2_ and liver cancer incidence, only So et al. found that an increase of NO_2_ concentration of 10 μg/m^3^ would affect the risk of liver cancer (HR = 1.17, 95%CI:1.02–1.35) (16, 20, 21). Therefore, to further explore whether there is an association between air pollution and the risk of primary liver cancer, we conducted a Mendelian randomization (MR) study using large-scale publicly available genome-wide association studies (GWAS) data with PM2.5, PM2.5–10, PM10, nitrogen dioxide and nitrogen oxides as exposures and primary liver cancer and its related biomarkers (Alpha-fetoprotein, Osteopontin, Glypican-3, and Arginase-1) as outcome to assess the causal relationship between air pollution and the risk of primary liver cancer.

## 2. Materials and methods

### 2.1. Study design

Our study is based on the Mendelian randomization design, which depends on three core assumptions that instrumental variants (1) are associated with the exposure, (2) are not associated with the outcome via a confounding pathway, and (3) do not affect the outcome directly, only possibly indirectly via the exposure. In this study, air pollution (PM2.5, PM2.5–10, PM10, nitrogen dioxide and nitrogen oxides) was used as the exposure factor, single nucleotide polymorphisms (SNPs) significantly related to air pollution were used as instrumental variables (IVs), and primary liver cancer and its related biomarkers (Alpha-fetoprotein, Osteopontin, Glypican-3, and Arginase-1) were the outcome variable. Here, we conducted a two-sample MR analysis to estimate the causal effects of air pollution and primary liver cancer. The flowchart of this Mendelian randomization study is presented in Supplementary Figure 1.

### 2.2. Data sources

The data sources are detailed in Table 1. We selected air pollution (including PM2.5, PM2.5–10, PM10, nitrogen dioxide and nitrogen oxides) as exposures, with data on all air pollution obtained from UK Biobank, a large prospective study with more than half a million United Kingdom participants for which data on phenotypes, genetic details, and genome-wide genotyping have been published (23, 24). We used the GWAS summary databases of air pollution for populations in Europe and East Asia. In European populations, the PM2.5 (GWAS ID: ukb-b-10,817), PM2.5–10 (GWAS ID: ukb-b-12,963), PM10 (GWAS ID: ukb-b-589), nitrogen dioxide (GWAS ID: ukb-b-2,618) and nitrogen oxides (GWAS ID: ukb-b-12,417) GWAS summary datasets included 423,796, 423,796, 455,314, 456,380, 456,380 participants, respectively. Among East Asian populations, the PM2.5 (GWAS ID: ukb-e-24006_EAS), PM2.5–10 (GWAS ID: ukb-e-24008_EAS), PM10 (GWAS ID: ukb-e-24005_EAS), nitrogen dioxide (GWAS ID: ukb-e-24016_EAS) and nitrogen oxides (GWAS ID: ukb-e-24004_EAS) GWAS summary datasets included 2,505, 2,505, 2,505, 2,625, 2,625 participants, respectively. Air pollution-related indicators were measured by land use regression (LUR) models (25).

**Table 1:** Summary of the genome-wide association studies (GWAS) included in this two-sample MR study.

Exposures/outcomes		Dataset	Sample size	Number of SNPs	Population	Consortium	Sex	Year
Particulate matter (PM)	PM2.5 um	ukb-b-10,817	423,796	9,851,867	European	MRC-IEU	Males and Females	2018
	PM2.5 um	ukb-e-24006_EAS	2,505	8,268,350	Asian (East Asia)	NA	Males and Females	2020
	PM2.5–10 um	ukb-b-12,963	423,796	9,851,867	European	MRC-IEU	Males and Females	2018
	PM2.5–10 um	ukb-e-24008_EAS	2,505	8,268,350	Asian (East Asia)	NA	Males and Females	2020
	PM10 um	ukb-b-589	455,314	9,851,867	European	MRC-IEU	Males and Females	2018
	PM10 um	ukb-e-24005_EAS	2,505	8,268,350	Asian (East Asia)	NA	Males and Females	2020
Nitrogen dioxide		ukb-b-2,618	456,380	9,851,867	European	MRC-IEU	Males and Females	2018
Nitrogen dioxide		ukb-e-24016_EAS	2,625	8,260,777	Asian (East Asia)	NA	Males and Females	2020
Nitrogen oxides		ukb-b-12,417	456,380	9,851,867	European	MRC-IEU	Males and Females	2018
Nitrogen oxides		ukb-e-24004_EAS	2,625	8,260,777	Asian (East Asia)	NA	Males and Females	2020
Primary liver cancer		finn-b-C3_LIVER_INTRAHEPATIC_BILE_DUCTS	218,752	16,380,466	European	NA	Males and Females	2021
Primary liver cancer		bbj-a-158	197,611	8,885,115	Asian (East Asia)	NA	Males and Females	2019
Alpha-fetoprotein		prot-a-53	3,301	10,534,735	European	NA	Males and Females	2018
Osteopontin		ebi-a-GCST90010244	1,322	18,221,494	European	NA	Males and Females	2020
Glypican-3		prot-c-4842_62_2	3,301	501,428	European	NA	Males and Females	2019
Arginase-1		ebi-a-GCST90010286	1,072	17,593,887	European	NA	Males and Females	2020

PM: Particulate matter; MR: Mendelian randomization; GWAS: Genome-wide association studies; SNPs: Single nucleotide polymorphisms; NA: No data.

We used primary liver cancer and its related biomarkers (Alpha-fetoprotein, Osteopontin, Glypican-3 and Arginase-1) as the outcome, and all GWAS data for primary liver cancer were obtained from FinnGen (European population) and Biobank Japan (East Asian population). The four biomarkers (only European population) Alpha-fetoprotein, Osteopontin, Glypican-3 and Arginase-1 were derived from the prot-a-53 (26), ebi-a-GCST90010244 (27), prot-c-4842_62_2 (28) and ebi-a-GCST90010286 (27) GWAS summary data. FinnGen is a large public-private research project that combines imputed genotype data generated from newly collected and legacy samples from the Finnish biobank and digital health record data from the Finnish Health Registry^1^ to provide new insights into disease genetics. As of August 2020, samples from 412,000 people have been collected and 224,737 have been analyzed, with 500,000 participants expected by the end of 20,232 (29, 30). Biobank Japan is a large patient-based biobank consisting of 200,000 patients. As a basic biobank for common disease gene research, the project has conducted genome-wide association studies for various diseases and identified many genetic variants associated with disease susceptibility and drug response. All publications are in this project from the project web site^2^ and open to the public (31). GWAS summary dataset of primary liver cancer in European populations (GWAS ID: finn-b-C3_LIVER_INTRAHEPATIC_BILE_DUCTS) contained 218,752 individuals (including 304 cases and 218,448controls). GWAS summary dataset of primary liver cancer in East Asian populations (GWAS ID: bbj-a-158) contained 197,611 individuals (including 1,866 cases and 195,745controls). Primary liver cancer cases were identified according to clinical diagnosis and conformed to the International Classification of Diseases, 8th Revision and 10th Revision codes.

### 2.3. Selection of instrumental variables

As shown in Supplementary Figure 1, in order to satisfy assumption 1, *p* < 5 × 10^−8^ was used as the genome-wide significance threshold for exposure, but only PM2.5 (European, ukb-b-10,817), PM10 (European, ukb-b-589), nitrogen dioxide (European, ukb-b-2,618) and nitrogen oxides (European, ukb-b-12,417) were able to pick out enough SNPs. Previous studies have shown that linear regression of each genetic variant on the risk factor with *p* < 5 × 10^−6^ as the screening criterion results in a low probability of weak instrumental variable bias in the MR Analysis (32, 33), so we lowered the genome-wide significance threshold of the remaining exposure (ukb-b-12,963, ukb-e-24006_EAS, ukb-e-24008_EAS, ukb-e-24005_EAS, ukb-e-24016_EAS and ukb-e-24004_EAS) to *p* < 5 × 10^−6^ to select enough SNPs as IVs associated with this significance level.

In order to remove SNPs with linkage disequilibrium (LD), *r*^2^ < 0.001 and kb > 10,000 was set when extracting IVs. If the selected SNP was not collected in the resulting GWAS, the proxy SNP in linkage disequilibrium (*r*^2^ > 0.8) was used. Palindromic SNPs were then removed to ensure that the effect of these SNPs on exposure corresponded to the same allele as the effect on outcome. Finally, we calculated the *R*^2^ (*R*^2^ = 2◊EAF◊(1–EAF)◊β^2^) (34) and *F*-statistic (*F* = *β*^2^/SE^2^) (35) for each SNP. *R*^2^ is the percentage of iron status variability explained by each SNP and *F* statistic to assess the presence of a weak IV bias. The *F*-statistic of each SNP we selected was > 10, suggesting that the genetic instruments selected strongly predicted the exposure (36). For specific SNP information and corresponding *R*^2^ and *F*-statistic, shown in Supplementary Tables 1–7.

### 2.4. Mendelian randomization analysis

To assess the causal relationship between air pollution and primary liver cancer and its related biomarkers, we used the inverse variance weighted (IVW) method to predict the genetic predictive value of the exposure factor for the outcome variable with an effect value of β. IVW can obtain an estimate of causal effect based on a single genetic IV through Wald ratio, and then select a fixed effect model to perform a meta-analysis of multiple estimates of causal effect based on a single gene IV, which can provide a reliable estimate of causal effect and is widely used in MR Analysis (32, 37). In order to further improve the reliability and accuracy of the study results, weighted median model, MR-Egger, simple model and weighted model methods were further used to verify the causal relationship between exposure factors and results, and were verified in both European and Asian populations (38). Biomarkers were only analyzed in European populations due to lack of GWAS data in East Asian populations.

### 2.5. Sensitivity analysis

First of all, we used leave-one-out method to test the sensitivity of the remaining SNPs after deleting SNPs one by one. If the results changed significantly, it indicated that the removed SNPs might be directly related to the results, which violated assumption 3 (39). Then, for the IVW method, Cochran’s Q test was used to evaluate the heterogeneity, and *p* > 0.05 indicated that there was no significant heterogeneity in the selected IVs (40). Finally, we need to perform pleiotropic tests using MR-Egger regression and MR-PRESSO global testing to ensure that IV does not influence the risk of primary liver cancer through other confounding factors or other biological pathways unrelated to air pollution exposure. The MR Egger regression effect model allows for causal estimation of pleiotropic effect corrections, evaluating instrumental intensity under the direct effect assumption independently of the null causality assumption, and MR-PRESSO enables a systematic assessment of the role of pleiotropy (41). The statistical threshold for IVs without pleiotropy was *p* > 0.05.

### 2.6. Statistical analysis

All analyses were performed using the packages “TwoSampleMR” (42) and “MR-PRESSO” (41) in R version 4.2.2. The threshold of statistical significance for evidence is *p* < 0.05.

## 3. Results

### 3.1. Air pollution and primary liver cancer

**Figure 1:**
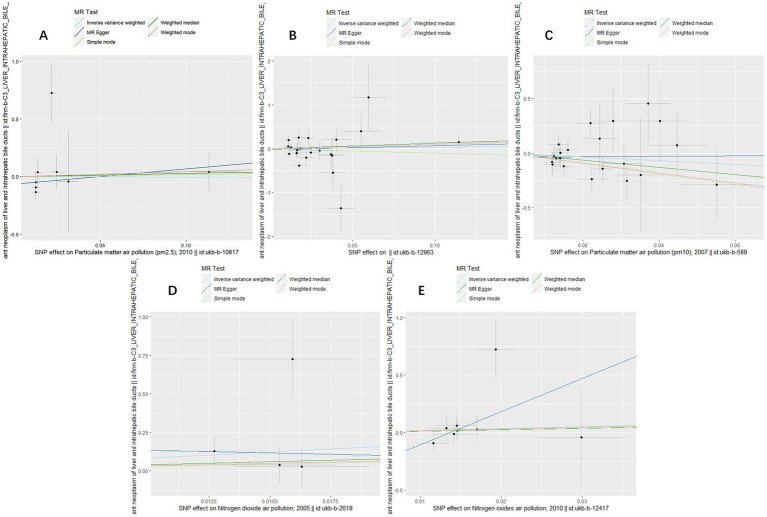
Scatter plots for causal single nucleotide polymorphism (SNP) effect of air pollution (particulate matter, nitrogen dioxide and nitrogen oxides) on primary liver cancer in European population. Each black point representing each SNP on the exposure (horizontal-axis) and on the outcome (vertical-axis) is plotted with error bars corresponding to each standard error (SE). The slope of each line corresponds to the combined estimate using each method of the inverse variance weighted (light blue line), the MR-Egger (blue line), the simple mode (light green line), the weighted median (green line), and the weighted mode (pink line). **(A)** PM2.5; **(B)** PM2.5–10; **(C)** PM10; **(D)** Nitrogen dioxide; **(E)** Nitrogen oxides.

**Figure 2:**
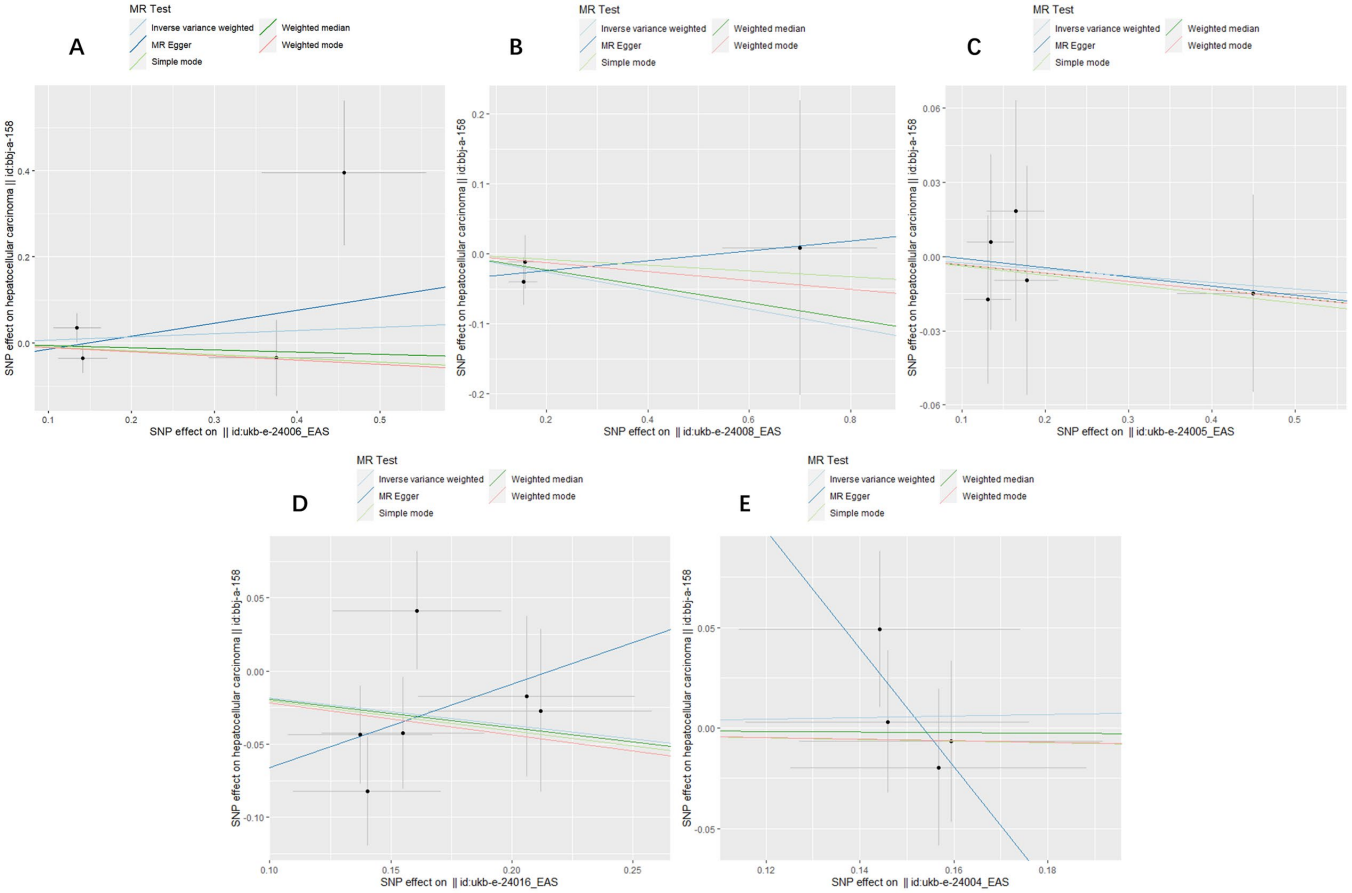
Scatter plots for causal SNP effect of air pollution (particulate matter, nitrogen dioxide and nitrogen oxides) on primary liver cancer in Asian population. Each black point representing each SNP on the exposure (horizontal-axis) and on the outcome (vertical-axis) is plotted with error bars corresponding to each standard error (SE). The slope of each line corresponds to the combined estimate using each method of the inverse variance weighted (light blue line), the MR-Egger (blue line), the simple mode (light green line), the weighted median (green line), and the weighted mode (pink line). **(A)** PM2.5; **(B)** PM2.5–10; **(C)** PM10; **(D)** Nitrogen dioxide; **(E)** Nitrogen oxides.

**Figure 3:**
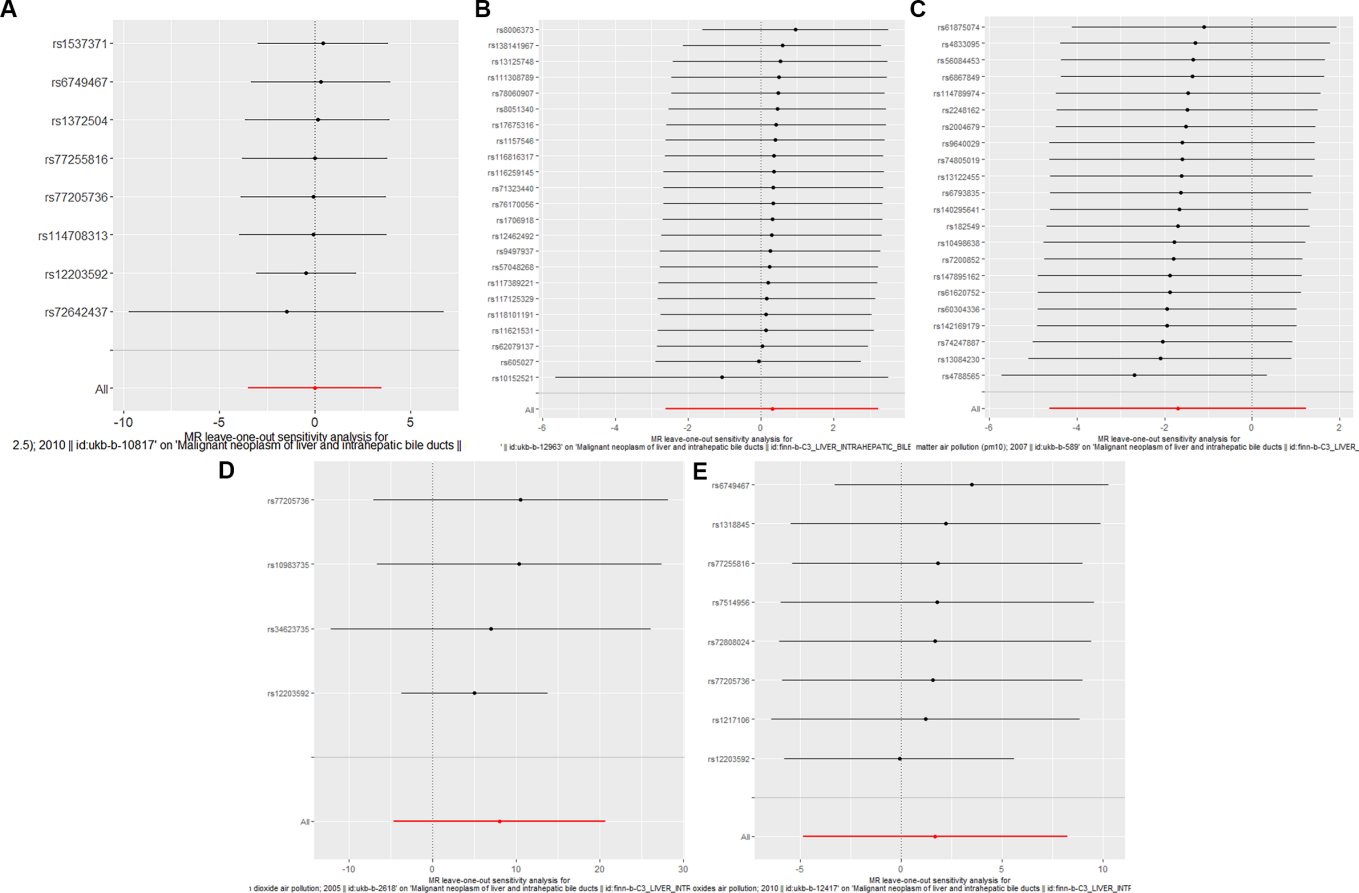
Forest plots of Leave-one-out analyses for causal SNP effect of air pollution (particulate matter, nitrogen dioxide and nitrogen oxides) on primary liver cancer in European population. The error bars indicate the 95% confidence interval (CI). **(A)** PM2.5; **(B)** PM2.5–10; **(C)** PM10; **(D)** Nitrogen dioxide; **(E)** Nitrogen oxides.

**Figure 4:**
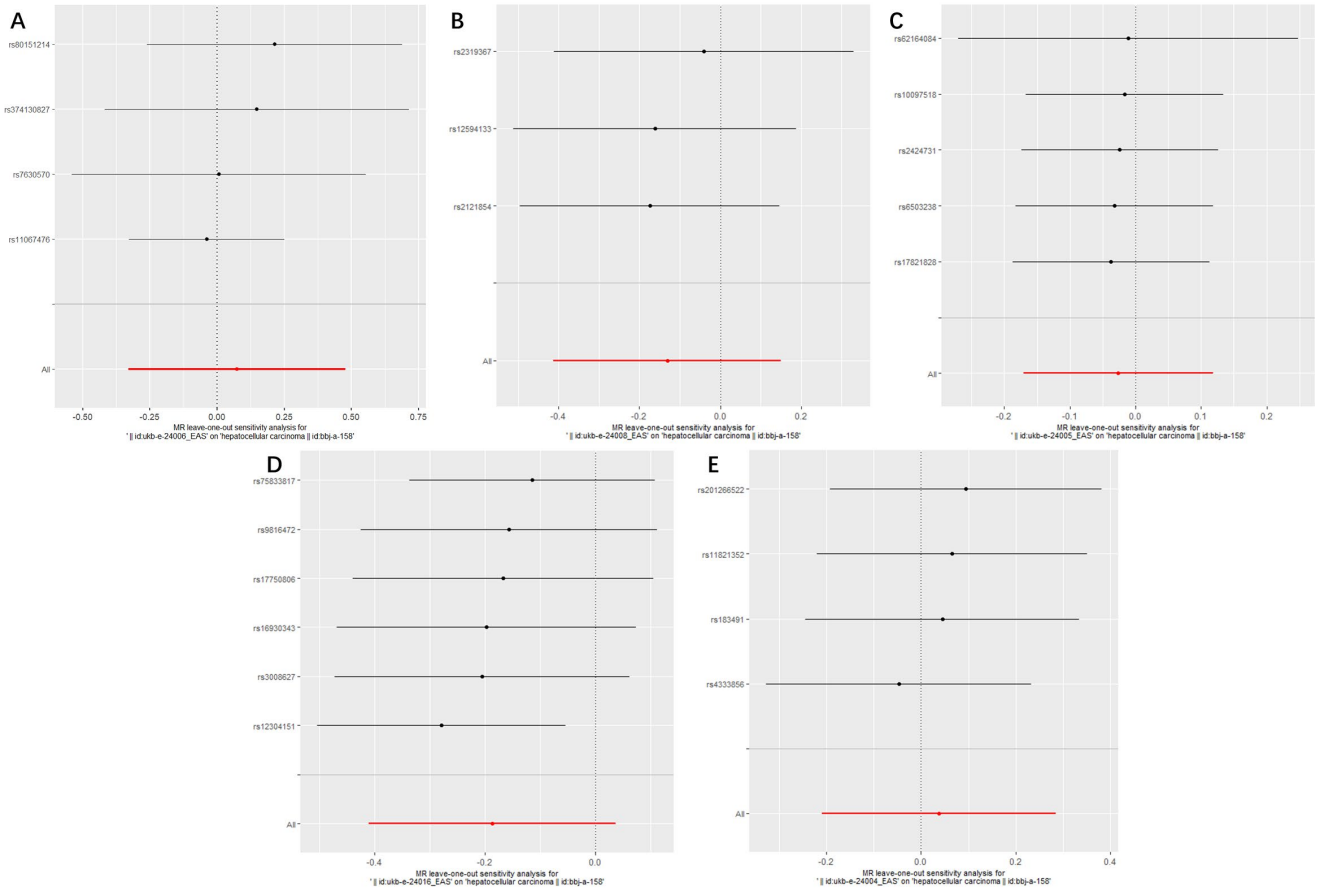
Forest plots of Leave-one-out analyses for causal SNP effect of air pollution (particulate matter, nitrogen dioxide and nitrogen oxides) on primary liver cancer in Asian population. The error bars indicate the 95% confidence interval (CI). **(A)** PM2.5; **(B)** PM2.5–10; **(C)** PM10; **(D)** Nitrogen dioxide; **(E)** Nitrogen oxides.

The MR results are shown in Table 2 (European population) and Table 3 (Asian population), as well as in Figure 1 (Scatter plots, European population), Figure 2 (Scatter plots, Asian population), Figure 3 (Forest plots, European population) and Figure 4 (Forest plots, Asian population).

**Table 2:** Mendelian randomization (MR) analysis of air pollution (particulate matter, nitrogen dioxide and nitrogen oxides, exposure) with primary liver cancer outcome in European population.

Exposures	Methods	Beta	*p*	Number of SNPs	*R* ^2^	*F*	*P* (Cochran’s Q heterogeneity test)	*P* (MR-Egger intercept test)	*P* (MR-PRESSO global test)
PM2.5	IVW	−0.014	0.993	8	0.069%	292.604	0.078	0.411	0.326	Weighted median	0.285	0.846	MR-Egger	1.315	0.596	Simple mode	0.191	0.955	Weighted mode	0.435	0.770
	PM2.5–10	IVW	0.313							0.833	23	0.130%	534.813	0.057	0.720	0.081	Weighted median	1.250	0.422	MR-Egger	0.879	0.691	Simple mode	−1.000	0.776	Weighted mode	0.990	0.522
		PM10	IVW							−1.698							0.257	22	0.159%	810.361	0.593	0.609	0.578	Weighted median	−3.305	0.107	MR-Egger	0.173	0.965	Simple mode	−4.763	0.182	Weighted mode	−4.506	0.171
			Nitrogen dioxide							IVW							6.478							0.215	4	0.032%	158.579	0.079	0.888	0.219	Weighted median	3.917	0.458	MR-Egger	−3.456	0.966	Simple mode	2.864	0.641	Weighted mode	3.270	0.594
										Nitrogen oxides							IVW							3.337							0.614	8	0.060%	283.730	0.204	0.198	0.271	Weighted median	1.283	0.720	MR-Egger	28.909	0.180	Simple mode	4.573	0.786	Weighted mode	4.237	0.730

PM, Particulate matter; MR, Mendelian randomization; IVW, Inverse variance weighted; SNPs, Single nucleotide polymorphisms.

*R*^2^: the percentage of iron status variability explained by each SNP; *F* statistic to assess the presence of a weak instrumental variable bias.

**Table 3:** Mendelian randomization (MR) analysis of air pollution (particulate matter, nitrogen dioxide and nitrogen oxides, exposure) with primary liver cancer outcome in Asian population (East Asia).

Exposures	Methods	Beta	*p*	Number of SNPs	*R*^2^	*F*	*P* (Cochran’s Q heterogeneity test)	*P* (MR-Egger intercept test)	*P* (MR-PRESSO global test)
PM2.5	IVW	0.074	0.718	4	3.527%	87.086	0.057	0.714	0.133
	Weighted median	−0.050	0.769						
	MR-Egger	0.300	0.660						
	Simple mode	−0.086	0.706						
	Weighted mode	−0.097	0.622						
PM2.5–10	IVW	−0.131	0.362	3	3.371%	72.691	0.740	0.675	NA
	Weighted median	−0.116	0.490						
	MR-Egger	0.070	0.885						
	Simple mode	−0.040	0.865						
	Weighted mode	−0.063	0.783						
PM10	IVW	−0.026	0.720	5	4.933%	112.709	0.512	0.936	0.984
	Weighted median	−0.033	0.701						
	MR-Egger	−0.037	0.814						
	Simple mode	−0.037	0.783						
	Weighted mode	−0.033	0.735						
Nitrogen dioxide	IVW	−0.186	0.101	6	5.536%	126.644	0.308	0.337	0.368
	Weighted median	−0.194	0.171						
	MR-Egger	0.569	0.463						
	Simple mode	−0.204	0.385						
	Weighted mode	−0.218	0.351						
Nitrogen oxides	IVW	0.038	0.760	4	4.014%	95.452	0.613	0.412	0.652
	Weighted median	−0.014	0.921						
	MR-Egger	−2.936	0.417						
	Simple mode	−0.040	0.857						
	Weighted mode	−0.038	0.853						

PM, Particulate matter; MR: Mendelian randomization; IVW, Inverse variance weighted; SNPs, Single nucleotide polymorphisms.

*R*^2^: the percentage of iron status variability explained by each SNP; *F* statistic to assess the presence of a weak instrumental variable bias.

To assess the causal effect of air pollution (including PM2.5, PM2.5–10, PM10, nitrogen dioxide, and nitrogen oxides) on primary liver cancer, we first performed MR Analysis in a European population. 8, 23, 22, 4, and 8 SNPs for PM2.5, PM2.5–10, PM10, nitrogen dioxide, and nitrogen oxides were identified after removal of chained unbalanced IVs (Supplementary Table 1). Using IVW, weighted median model, MR-Egger, simple model and weighted model methods, we found no evidence of a causal relationship between air pollution and primary liver cancer risk (IVW method, PM2.5: *p* = 0.993; PM2.5–10: *p* = 0.833; PM10: *p* = 0.257; nitrogen dioxide: *p* = 0.215; nitrogen oxides: *p* = 0.614), and there was no evidence of significant heterogeneity or horizontal pleiotropy (Table 2). The scatter plots of the causal relationships between air pollution and the risk of primary liver cancer are shown in Figure 1. Leave-one-out analyses (Figure 3) showed that removing each SNP in turn had little effect on the results, suggesting that no single SNP had a significant effect on the overall causal effect estimates.

The MR analysis was repeated in the East Asian population to enhance the confidence of the above results. 4, 3, 5, 6, and 4 SNPs for PM2.5, PM2.5–10, PM10, nitrogen dioxide, and nitrogen oxides were identified after removal of chained unbalanced IVs (Supplementary Table 2). Consistent with the findings in the European population, we found no causal relationship between air pollution and primary liver cancer risk in the East Asian population using the above five methods (IVW method, PM2.5: *p* = 0.718; PM2.5–10: *p* = 0.362; PM10: *p* = 0.720; nitrogen dioxide: *p* = 0.101; nitrogen oxides: *p* = 0.760), and no significant heterogeneity and pleiotropy were found (Table 3). The scatter plots are shown in Figure 2. Leave-one-out analyses also did not identify abnormal SNPs (Figure 4).

### 3.2. Air pollution and biomarkers

The MR results are shown in Table 4, as well as in Supplementary Figures 2–5 (Scatter plots), and Supplementary Figures 6–9 (Forest plots).

**Table 4:** Mendelian randomization (MR) analysis of air pollution (particulate matter, nitrogen dioxide and nitrogen oxides, exposure) with biomarkers in primary liver cancer in European population (IVW method).

Exposures	Outcomes (biomarkers)	Beta (95%CI)	*p*	Number of SNPs	*R*^2^	*F*	*P* (Cochran’s Q heterogeneity test)	*P* (MR-Egger intercept test)	*P* (MR-PRESSO global test)
PM2.5	Alpha-fetoprotein	−0.64 (−2.05, 0.76)	0.370	7	0.059%	257.485	0.871	0.287	0.884	Osteopontin	−0.35 (−2.10, 1.40)	0.695	8	0.069%	292.604	0.684	0.177	0.671	Glypican-3	−2.12 (−5.58,1.33)	0.228	4	0.038%	166.694	0.691	0.464	0.725	Arginase-1	1.39 (−0.49, 3.29)	0.147	8	0.069%	292.604	0.631	0.360	0.663
PM2.5–10	Alpha-fetoprotein	−0.50 (−1.82, 0.80)	0.405	21	0.117%	482.903	0.106	0.464	0.091	Osteopontin	0.20 (−1.26, 1.68)	0.780	23	0.129%	531.943	0.155	0.214	0.187	Glypican-3	−3.30 (−6.59, 0.02)	0.058	6	0.033%	136.070	0.575	0.790	0.592	Arginase-1	−0.41 (−1.80, 0.97)	0.559	23	0.129%	531.943	0.794	0.890	0.810
PM10	Alpha-fetoprotein	0.08 (−0.77, 0.95)	0.842	22	0.159%	810.360	0.506	0.955	0.515	Osteopontin	0.49 (−0.99, 1.98)	0.517	22	0.159%	810.360	0.882	0.656	0.887	Glypican-3	−0.39 (−3.69, 2.90)	0.814	6	0.052%	271.045	0.125	0.579	0.180	Arginase-1	1.78 (−0.08, 3.64)	0.061	22	0.159%	810.360	0.136	0.070	0.127
Nitrogen dioxide	Alpha-fetoprotein	0.51 (−1.33, 2.36)	0.585	5	0.039%	191.857	0.343	0.530	0.391	Osteopontin	0.86 (−2.50, 4.24)	0.615	5	0.039%	191.857	0.856	0.708	0.855	Glypican-3	−1.47 (−4.70, 1.75)	0.388	5	0.039%	191.857	0.625	0.398	0.654	Arginase-1	−0.44 (−4.10, 3.21)	0.812	5	0.039%	191.857	0.432	0.195	0.494
Nitrogen oxides	Alpha-fetoprotein	0.33 (−1.11, 1.78)	0.652	8	0.060%	283.730	0.394	0.527	0.430	Osteopontin	−1.52 (−4.24, 1.19)	0.271	8	0.060%	283.730	0.833	0.286	0.819	Glypican-3	−1.56(−5.26, 2.12)	0.405	4	0.033%	160.302	0.682	0.458	0.702	Arginase-1	4.46 (0.83, 8.08)	0.015	5	0.033%	155.317	0.453	0.366	0.502

PM, Particulate matter; MR, Mendelian randomization; IVW, Inverse variance weighted; SNPs, Single nucleotide polymorphisms; CI, Confidence Interval.

*R*^2^: the percentage of iron status variability explained by each SNP; *F* statistic to assess the presence of a weak instrumental variable bias; Genetic predictive value of the exposure factor for the outcome variable with an effect value of Beta.

In order to further verify the causal relationship between air pollution and primary liver cancer, we selected four biomarkers (Alpha-fetoprotein, Osteopontin, Glypican-3 and Arginase-1) which are closely related to primary liver cancer as the outcome and conducted MR Analysis again. Consistent with the above results, we did not find any causal association between air pollution and Alpha-fetoprotein (PM2.5: *p* = 0.370; PM2.5–10: *p* = 0.405; PM10: *p* = 0.842; nitrogen dioxide: *p* = 0.585; nitrogen oxides: *p* = 0.652), Osteopontin (PM2.5: *p* = 0.695; PM2.5–10: *p* = 0.780; PM10: *p* = 0.517; nitrogen dioxide: *p* = 0.615; nitrogen oxides: *p* = 0.271) and Glypican-3 (PM2.5: *p* = 0.228; PM2.5–10: *p* = 0.058; PM10: *p* = 0.814; nitrogen dioxide: *p* = 0.388; nitrogen oxides: *p* = 0.405) through IVW method. The results showed no heterogeneity or pleiotropy (Table 4).

It is worth mentioning that we initially extracted 8 SNPs (rs1217106, rs12203592, rs1318845, rs6749467, rs72808024, rs7514956, rs77205736, and rs77255816) that were strongly associated with nitrogen oxides as instrumental variables and found a significant association between nitrogen oxides and Arginase-1 using IVW method (Beta: 3.56; 95%CI: 0.63–6.49; *p* = 0.017). Subsequently, we searched these 8 SNPs for possible confounding related to primary liver cancer through the PhenoScanner database (http://www.Phenoscanner.medschl.Cam.ac.uk/ accessed on March 12, 2023) one by one, and found 3 SNPs (rs1217106, rs12203592 and rs77205736) related to alcohol intake frequency, cancers (breast cancer, nonmelanoma skin cancer, cutaneous squamous cell carcinoma, and basal cell carcinoma) and Body mass index (BMI). After deleting them, we analyzed again and found that the results were still statistically significant (Beta: 4.46; 95%CI: 0.83–8.08; *p* = 0.015), and there was no heterogeneity or pleiotropy. The specific SNP information, corresponding *R*^2^ and *F*-statistic are shown in Supplementary Tables 3–7.

## 4. Discussion

This study is the first to evaluate the causal relationship between air pollution (including PM2.5, PM2.5–10, PM10, nitrogen dioxide and nitrogen oxides) and primary liver cancer using MR methods. We found no genetic evidence of an association between air pollution and primary liver cancer risk in either the European or East Asian populations. However, in a further MR analysis of air pollution and biomarkers associated with primary liver cancer (Alpha-fetoprotein, Osteopontin, Glypican-3 and Arginase-1), we found a significant association between nitrogen oxides and biomarker Arginase-1 related to hepatocellular differentiation (Beta: 3.56; 95%CI: 0.63–6.49; *p* = 0.017), which remained statistically significant after adjustment (Beta: 4.46; 95%CI: 0.83–8.08; *p* = 0.015) for possible confounding alcohol intake frequency, body mass index (BMI) and cancers (breast cancer, nonmelanoma skin cancer, cutaneous squamous cell carcinoma, and basal cell carcinoma).

Our findings further confirm several population-based cohort studies. Marie Pedersen et al. (20) pooled data from 174,770 participants in four cohorts [Diet, Cancer and Health study (43), Vorarlberg Health Monitoring and Promotion Program (44), European Prospective Investigation into Cancer and Nutrition (EPIC)-Varese (45) and EPIC-Turin (46)] from European Study of Cohorts for Air Pollution Effects (ESCAPE) in Denmark, Austria, and Italy to examine the association between air pollution (including PM2.5, PM2.5–10, PM10, nitrogen dioxide and nitrogen oxides) and the risk of primary liver cancer, and land-use regression models were used to measure PM2.5, PM2.5–10, PM10, nitrogen dioxide and nitrogen oxides. During an average follow-up period of 17 years, 279 patients with liver cancer were diagnosed, and the meta-analysis found that PM2.5, PM2.5–10, PM10, nitrogen dioxide and nitrogen oxides were all associated with an increased incidence of primary liver cancer, with Hazard ratios (HRs) greater than 1 for all exposures, but none of the associations were statistically significant. After adjusting for age, sex, smoking, drinking, high-risk occupations and other confounding factors that might be associated with primary liver cancer, no statistical association was found between air pollution and primary liver cancer. Unlike our findings, another prospective cohort study in Taiwan (19) followed 23,820 participants without a history of liver cancer for an average of 16.9 years suggested that every 0.73 μg/m^3^ increase in PM 2.5 increased the risk of hepatocellular carcinoma incidence by 22% in Penghu Islets (HR = 1.22, 95%CI: 1.02–1.47). However, PM2.5 levels in Taiwan and the Main Island of Taiwan (per 1 and 13.1 μg/m^3^) were not statistically associated with the risk of liver cancer. The reasons for the two conclusions may be related to the different levels of air pollution in different areas or the different susceptibility of people in different areas to air pollution, and genetic factors may also play a role. Therefore, we selected SNPs strongly associated with air pollution as instrumental variables, used two-sample Mendelian randomization to conduct causal analysis on air pollution and primary liver cancer at the genetic level, and verified them separately on European and East Asian populations, so as to improve the reliability and credibility of our research conclusions. Our study demonstrates the lack of statistical causality between air pollution and primary liver cancer, reduces the possibility of their clinical relevance, refutes the role of air pollution in the etiology of primary liver cancer, and complements and updates the methodology of several cohort studies that have reached similar conclusions as our study.

Alpha-fetoprotein (AFP), Osteopontin (OPN) and Glypican-3 (GPC-3) are commonly used as tumor markers for primary liver cancer. AFP, a glycoprotein derived from embryonic endodermal cells, is an important cytokine closely related to the malignant growth of tumors, which can promote the malignant transformation of hepatocytes and the occurrence and development of liver cancer and up to 70% of patients with liver cancer have elevated serum AFP levels (47). AFP has been reported to be 52% sensitive to tumors larger than 3 cm in diameter in hepatocellular carcinoma patients, and is the most widely used tumor marker in clinical practice (48). OPN, a highly modified extracellular matrix protein, is found in 0% of serum in healthy people and increases in hepatitis, cirrhosis and liver cancer patients. OPN even outperforms AFP in distinguishing cirrhosis from liver cancer. When OPN and AFP are combined in the diagnosis of hepatocellular carcinoma, the sensitivity can increase to 65% (6, 49). GPC-3 is a hepatocellular carcinoma related biomarker with a specificity of up to 97%, which can detect hepatocellular carcinoma at an earlier stage than AFP and one study showed that serum GPC-3 levels in 50% of patients with early hepatocellular carcinoma were > 300 ng/L, despite their serum AFP levels < 100 μg/L (50). MR Analysis of air pollution and primary liver cancer related tumor markers AFP, OPN and GPC-3 found no causal association, further confirming our previous conclusion that there was no statistical association between air pollution and primary liver cancer risk.

Arginase-1 (Arg-1), an enzyme that catalyzes the hydrolysis of arginine to ornithine and urea in the urea cycle, is mainly expressed in the cytoplasm of hepatocytes and is not expressed in bile duct epithelial cells, Kupffer cells, or vascular endothelial cells, and thus can be used in the differential diagnosis between hepatocellular carcinoma and other potentially confounding malignancies. Arg-1 is reported to be a highly specific biomarker for hepatocellular differentiation, with sensitivities of 100, 96.2 and 85.7% in highly differentiated, moderately differentiated and poorly differentiated hepatocellular carcinoma, respectively (51, 52). Our results found a significant association between nitrogen oxides and Arginase-1, a biomarker highly associated with hepatocellular differentiation, which remained statistically significant after adjusting for possible confounding factors such as alcohol intake frequency, body mass index (BMI) and cancers (breast cancer, nonmelanoma skin cancer, cutaneous squamous cell carcinoma, and basal cell carcinoma; Beta: 4.46; 95%CI: 0.83–8.08; *p* = 0.015). Currently, there is a lack of relevant research on the effects of nitrogen oxides on Arg-1, but it has been shown that nitric oxide can affect liver cell differentiation by affecting the tumor microenvironment. On the one hand, nitric oxide can play a role in tumor differentiation, growth progression and metastasis by modulating the expression of multiple inflammatory factors. On the other hand, it can affect and regulate anabolism and catabolism, including sugar, fatty acid and amino acid metabolism, to affect the tumor microenvironment, and which plays a very important role in the hepatocellular differentiation and the conversion of normal cells into tumor cells, and even determines to some extent the direction and type of differentiation of liver cancer (53–56). To some extent, this may explain our results that nitrogen oxides air pollution may affect hepatocellular differentiation by altering the hepatocyte microenvironment, but further *in vivo* and *in vitro* experiments are needed to confirm this hypothesis.

There were several limitations to our study. To begin with, although we conducted the MR Analysis on the causal relationship between air pollution and primary liver cancer in both European and East Asian populations, due to the limitation of data, we only analyzed the European population when we analyzed the tumor markers (Alpha-fetoprotein, Osteopontin, Glypican-3 and Arginase-1) of liver cancer, and whether this relationship is also present in other populations needs more verification. Additionally, our results in East Asian populations were based on a 5 × 10^−6^ significance level, as there were not enough SNPs associated with a 5 × 10^−8^ genome-wide significance threshold, and this may require an expanded sample size to further validate our conclusions.

## 5. Conclusion

In conclusion, our results suggested that there is no causal association between air pollution (including PM2.5, PM2.5–10, PM10, nitrogen dioxide, and nitrogen oxides) and primary liver cancer. However, there was a statistical association between nitrogen oxides and Arg-1, further experimental and mechanistic studies are needed to verify the validity of the findings obtained in this study.

## Data availability statement

Publicly available datasets were analyzed in this study. This data can be found at: https://gwas.mrcieu.ac.uk/datasets/.

## Author contributions

MS and MG: designing, carrying out the study, analyzing the data, and writing the article. ML, TW, and TZ: carrying out the study, and revising. JQ: designing, revising, and financial support. All authors contributed to the article and approved the submitted version.

## Funding

This work was supported by the National Natural Science Foundation Program of China (82073653 and 81803313), Hunan Outstanding Youth Fund Project (2022JJ10087), National Key Research and Development Project (2018YFE0114500), China Postdoctoral Science Foundation (2020M682644), Hunan Provincial Science and Technology Talent Support Project (2020TJ-N07), Hunan Provincial Key Research and Development Program (2018SK2063), Open Project from NHC Key Laboratory of Birth Defect for Research and Prevention (KF2020006), Natural Science Foundation of Hunan Province (2018JJ2551), Natural Science Foundation of Hunan Province of China (2022JJ40207), and Changsha Municipal Natural Science Foundation (kq2202470).

## Conflict of interest

The authors declare that the study was conducted in the absence of any commercial or financial relationships that could be construed as a potential conflict of interest.

## Publisher’s note

All claims expressed in this article are solely those of the authors and do not necessarily represent those of their affiliated organizations, or those of the publisher, the editors and the reviewers. Any product that may be evaluated in this article, or claim that may be made by its manufacturer, is not guaranteed or endorsed by the publisher.

## Footnotes

^1^https://www.FinnGen.fi\/en

^2^https://biobankjp.org//work\/public.html

## Abbreviations

PM: particulate matter
NO_2_: nitrogen dioxide
NO_x_: nitrogen oxides
IVs: instrumental variables
MR: Mendelian randomization
GWAS: genome-wide association studies
IVW: inverse variance weighted
SNPs: single nucleotide polymorphisms
BMI: body mass index
AFP: alpha-fetoprotein
OPN: osteopontin
GPC-3: Glypican-3
Arg-1: Arginase-1
CI: confidence interval
HR: hazard ratio
