# Supplementary material for: Association between air pollution and primary liver cancer in European and east Asian populations: a Mendelian randomization study
Source: Front Public Health. 2023 Jul 27;11:1212301. doi: 10.3389/fpubh.2023.1212301 (PMC10415013; doi:10.3389/fpubh.2023.1212301)
Supplement: Supplementary file 1 [file Data_Sheet_1.docx]

Supplementary Material

## Supplementary Tables

**Supplementary Table 1.** Genetic variants significantly associated with air pollution exposure as instrumental variables and primary liver cancer as outcomes in European population.

| SNP | Effect allele | Other allele | Beta | SE | EAF | *P* | F | R^2^ |
| --- | --- | --- | --- | --- | --- | --- | --- | --- |
| PM2.5: | | | | | | | | |
| rs114708313 | T | A | 0.024558 | 0.004478 | 0.06585 | 4.20E-08 | 30.07625 | 7.41973E-05 |
| rs12203592 | T | C | 0.021666 | 0.002591 | 0.212894 | 6.20E-17 | 69.91813 | 0.000157322 |
| rs1372504 | A | G | 0.012291 | 0.002219 | 0.374311 | 3.10E-08 | 30.67374 | 7.07659E-05 |
| rs1537371 | A | C | 0.012371 | 0.002149 | 0.500143 | 8.50E-09 | 33.14877 | 7.65146E-05 |
| rs6749467 | A | G | -0.01239 | 0.002183 | 0.465814 | 1.40E-08 | 32.22849 | 7.64207E-05 |
| rs72642437 | T | C | 0.113396 | 0.019135 | 0.003862 | 3.10E-09 | 35.11871 | 9.89367E-05 |
| rs77205736 | T | C | 0.013522 | 0.002413 | 0.273909 | 2.10E-08 | 31.39913 | 7.27282E-05 |
| rs77255816 | T | C | 0.031394 | 0.005728 | 0.036507 | 4.20E-08 | 30.04086 | 6.9333E-05 |
| PM2.5-10: | | | | | | | | |
| rs10152521 | C | T | 0.114394 | 0.023431 | 0.002527 | 1.00E-06 | 23.83512 | 6.60E-05 |
| rs111308789 | A | T | -0.02194 | 0.004729 | 0.056635 | 3.50E-06 | 21.52391 | 5.14E-05 |
| rs1157546 | C | T | -0.0246 | 0.004901 | 0.053047 | 5.20E-07 | 25.20478 | 6.08E-05 |
| rs11621531 | A | G | -0.02313 | 0.004487 | 0.063928 | 2.50E-07 | 26.56476 | 6.40E-05 |
| rs116259145 | A | C | 0.030164 | 0.006185 | 0.031996 | 1.10E-06 | 23.78444 | 5.64E-05 |
| rs116816317 | A | G | 0.037649 | 0.007965 | 0.019771 | 2.30E-06 | 22.34258 | 5.49E-05 |
| rs117125329 | G | C | 0.055167 | 0.011992 | 0.008295 | 4.20E-06 | 21.16166 | 5.01E-05 |
| rs117389221 | C | T | -0.04022 | 0.008501 | 0.017253 | 2.20E-06 | 22.38495 | 5.49E-05 |
| rs118101191 | T | G | 0.059791 | 0.011063 | 0.010103 | 6.50E-08 | 29.20813 | 7.15E-05 |
| rs12462492 | T | G | -0.01239 | 0.002496 | 0.257064 | 6.90E-07 | 24.64175 | 5.86E-05 |
| rs13125748 | A | C | 0.011453 | 0.002502 | 0.257878 | 4.70E-06 | 20.95909 | 5.02E-05 |
| rs138141967 | T | G | -0.04294 | 0.009155 | 0.01498 | 2.70E-06 | 21.99721 | 5.44E-05 |
| rs1706918 | A | G | 0.018349 | 0.003677 | 0.098932 | 6.00E-07 | 24.90639 | 6.00E-05 |
| rs17675316 | G | A | 0.036905 | 0.00788 | 0.021631 | 2.80E-06 | 21.93191 | 5.76E-05 |
| rs57048268 | C | A | -0.01102 | 0.002365 | 0.311303 | 3.20E-06 | 21.70542 | 5.21E-05 |
| rs605027 | T | C | -0.01139 | 0.002454 | 0.729529 | 3.40E-06 | 21.56452 | 5.12E-05 |
| rs62079137 | C | T | -0.01735 | 0.003743 | 0.107445 | 3.60E-06 | 21.49039 | 5.77E-05 |
| rs71323440 | T | C | 0.016293 | 0.003408 | 0.114944 | 1.70E-06 | 22.85868 | 5.40E-05 |
| rs76170056 | A | C | 0.016508 | 0.003462 | 0.110936 | 1.90E-06 | 22.73626 | 5.38E-05 |
| rs78060907 | A | C | -0.03805 | 0.007698 | 0.020337 | 7.70E-07 | 24.43949 | 5.77E-05 |
| rs8006373 | A | T | -0.01758 | 0.003778 | 0.091464 | 3.30E-06 | 21.65915 | 5.14E-05 |
| rs8051340 | G | C | 0.016073 | 0.003326 | 0.122584 | 1.30E-06 | 23.35649 | 5.56E-05 |
| rs9497937 | A | C | -0.01277 | 0.002577 | 0.232437 | 7.20E-07 | 24.55559 | 5.82E-05 |
| PM10: | | | | | | | | |
| rs10498638 | C | T | 0.014014 | 0.002537 | 0.188322 | 3.30E-08 | 30.51287 | 6.00E-05 |
| rs114789974 | A | C | -0.05521 | 0.009635 | 0.010467 | 1.00E-08 | 32.83232 | 6.31E-05 |
| rs13084230 | T | C | -0.01356 | 0.00246 | 0.200319 | 3.50E-08 | 30.39524 | 5.89E-05 |
| rs13122455 | T | C | -0.014 | 0.002464 | 0.199995 | 1.30E-08 | 32.29318 | 6.28E-05 |
| rs140295641 | A | T | -0.03514 | 0.006172 | 0.027357 | 1.30E-08 | 32.40604 | 6.57E-05 |
| rs142169179 | A | G | 0.040185 | 0.00734 | 0.020279 | 4.40E-08 | 29.97094 | 6.42E-05 |
| rs147895162 | C | T | -0.04476 | 0.008107 | 0.015135 | 3.40E-08 | 30.48071 | 5.97E-05 |
| rs182549 | T | C | -0.01242 | 0.002219 | 0.738828 | 2.10E-08 | 31.35735 | 5.96E-05 |
| rs2004679 | C | T | 0.011915 | 0.002138 | 0.307685 | 2.50E-08 | 31.04924 | 6.05E-05 |
| rs2248162 | C | T | 0.011819 | 0.002047 | 0.63988 | 7.80E-09 | 33.33078 | 6.44E-05 |
| rs4788565 | A | G | -0.02192 | 0.003994 | 0.066776 | 4.10E-08 | 30.11083 | 5.99E-05 |
| rs4833095 | C | T | 0.025111 | 0.002406 | 0.206945 | 1.70E-25 | 108.9646 | 0.000207 |
| rs56084453 | G | A | 0.014935 | 0.002412 | 0.209746 | 5.90E-10 | 38.35178 | 7.39E-05 |
| rs60304336 | T | G | 0.027932 | 0.00503 | 0.040906 | 2.80E-08 | 30.84191 | 6.12E-05 |
| rs61620752 | G | T | 0.016069 | 0.002767 | 0.148343 | 6.40E-09 | 33.71969 | 6.52E-05 |
| rs61875074 | C | A | 0.022266 | 0.003841 | 0.073029 | 6.80E-09 | 33.6006 | 6.71E-05 |
| rs6793835 | A | G | -0.01299 | 0.002238 | 0.263796 | 6.60E-09 | 33.66108 | 6.55E-05 |
| rs6867849 | T | A | -0.03148 | 0.005232 | 0.040477 | 1.80E-09 | 36.1985 | 7.70E-05 |
| rs7200852 | A | C | -0.02443 | 0.00445 | 0.054732 | 4.00E-08 | 30.12939 | 6.17E-05 |
| rs74247887 | T | C | 0.037132 | 0.005881 | 0.028721 | 2.70E-10 | 39.86984 | 7.69E-05 |
| rs74805019 | C | G | -0.03071 | 0.005482 | 0.033712 | 2.10E-08 | 31.38607 | 6.15E-05 |
| rs9640029 | T | C | -0.0138 | 0.001974 | 0.478233 | 2.70E-12 | 48.89785 | 9.51E-05 |
| Nitrogen dioxide: | | | | | | | | |
| rs10983735 | A | G | 0.016311 | 0.002803 | 0.154461 | 5.90E-09 | 33.86681 | 6.95E-05 |
| rs12203592 | T | C | 0.015941 | 0.002408 | 0.219436 | 3.60E-11 | 43.82627 | 8.71E-05 |
| rs34623735 | T | C | 0.01272 | 0.002157 | 0.33449 | 3.70E-09 | 34.7748 | 7.20E-05 |
| rs77205736 | T | C | 0.015406 | 0.002269 | 0.274855 | 1.10E-11 | 46.11078 | 9.46E-05 |
| Nitrogen oxides: | | | | | | | | |
| rs1217106 | G | A | 0.014557 | 0.002519 | 0.782419 | 7.50E-09 | 33.40472 | 7.22E-05 |
| rs12203592 | T | C | 0.01937 | 0.002457 | 0.219436 | 3.20E-15 | 62.16427 | 0.000129 |
| rs1318845 | C | T | -0.01417 | 0.002596 | 0.200795 | 4.80E-08 | 29.80228 | 6.45E-05 |
| rs6749467 | A | G | -0.01166 | 0.002096 | 0.46467 | 2.60E-08 | 30.95781 | 6.77E-05 |
| rs72808024 | C | A | -0.01703 | 0.002906 | 0.148435 | 4.60E-09 | 34.33572 | 7.33E-05 |
| rs7514956 | C | A | -0.01461 | 0.002652 | 0.186513 | 3.60E-08 | 30.34155 | 6.48E-05 |
| rs77205736 | T | C | 0.013265 | 0.002315 | 0.274855 | 1.00E-08 | 32.84429 | 7.01E-05 |
| rs77255816 | T | C | 0.029905 | 0.005471 | 0.036924 | 4.60E-08 | 29.87975 | 6.36E-05 |

**Supplementary Table 2.** Genetic variants significantly associated with air pollution exposure as instrumental variables and primary liver cancer as outcomes in Asian population.

| SNP | Effect allele | Other allele | Beta | SE | EAF | *P* | F | R^2^ |
| --- | --- | --- | --- | --- | --- | --- | --- | --- |
| PM2.5: | | | | | | | | |
| rs11067476 | C | T | 0.4566 | 0.09914 | 0.97903 | 4.12E-06 | 21.21163 | 0.00856 |
| rs374130827 | A | G | -0.3753 | 0.08189 | 0.96941 | 4.59E-06 | 21.00368 | 0.008354 |
| rs7630570 | A | G | -0.1344 | 0.02863 | 0.4434 | 2.69E-06 | 22.03717 | 0.008916 |
| rs80151214 | G | A | -0.1413 | 0.02957 | 0.6162 | 1.77E-06 | 22.83398 | 0.009444 |
| PM2.5-10: | | | | | | | | |
| rs12594133 | G | A | -0.1576 | 0.0335 | 0.7705 | 2.56E-06 | 22.13211 | 0.008784 |
| rs2121854 | G | A | 0.7002 | 0.1528 | 0.0135 | 4.61E-06 | 20.99895 | 0.013059 |
| rs2319367 | T | C | 0.1543 | 0.02838 | 0.5273 | 5.39E-08 | 29.56018 | 0.011869 |
| PM10: | | | | | | | | |
| rs10097518 | G | A | -0.1311 | 0.0286 | 0.3766 | 4.58E-06 | 21.01229 | 0.00807 |
| rs17821828 | T | G | -0.1644 | 0.03431 | 0.7724 | 1.66E-06 | 22.95949 | 0.009503 |
| rs2424731 | C | T | -0.1775 | 0.03822 | 0.1728 | 3.40E-06 | 21.56827 | 0.009007 |
| rs62164084 | C | T | -0.4495 | 0.09072 | 0.96386 | 7.24E-07 | 24.5501 | 0.014076 |
| rs6503238 | C | T | -0.1345 | 0.02828 | 0.3995 | 1.97E-06 | 22.61964 | 0.00868 |
| Nitrogen dioxide: | | | | | | | | |
| rs12304151 | T | A | 0.1608 | 0.03486 | 0.7884 | 3.97E-06 | 21.27734 | 0.008627 |
| rs16930343 | A | G | 0.2118 | 0.04618 | 0.8859 | 4.50E-06 | 21.03507 | 0.009069 |
| rs17750806 | C | T | -0.1551 | 0.0337 | 0.7092 | 4.19E-06 | 21.18185 | 0.009922 |
| rs3008627 | C | A | -0.2061 | 0.04487 | 0.1162 | 4.35E-06 | 21.09812 | 0.008725 |
| rs75833817 | A | C | 0.1402 | 0.03069 | 0.4457 | 4.94E-06 | 20.86903 | 0.009712 |
| rs9816472 | C | T | -0.1372 | 0.02981 | 0.5532 | 4.16E-06 | 21.18284 | 0.009305 |
| Nitrogen oxides: | | | | | | | | |
| rs11821352 | G | T | -0.1595 | 0.03234 | 0.7571 | 8.15E-07 | 24.32436 | 0.009357 |
| rs183491 | T | C | -0.1459 | 0.03024 | 0.3335 | 1.39E-06 | 23.27807 | 0.009463 |
| rs201266522 | A | G | 0.1568 | 0.03155 | 0.4978 | 6.66E-07 | 24.6998 | 0.012293 |
| rs4333856 | A | G | -0.1442 | 0.02997 | 0.6813 | 1.49E-06 | 23.15032 | 0.00903 |

**Supplementary Table 3**. Genetic variants significantly associated with PM2.5 exposure as instrumental variables and biomarkers as outcomes in European population.

| SNP | Effect allele | Other allele | Beta | SE | EAF | *P* | F | R^2^ |
| --- | --- | --- | --- | --- | --- | --- | --- | --- |
| Alpha-fetoprotein: | | | | | | | | |
| rs114708313 | T | A | 0.024558 | 0.004478 | 0.06585 | 4.20E-08 | 30.07625 | 7.41973E-05 |
| rs12203592 | T | C | 0.021666 | 0.002591 | 0.212894 | 6.20E-17 | 69.91813 | 0.000157322 |
| rs1372504 | A | G | 0.012291 | 0.002219 | 0.374311 | 3.10E-08 | 30.67374 | 7.07659E-05 |
| rs1537371 | A | C | 0.012371 | 0.002149 | 0.500143 | 8.50E-09 | 33.14877 | 7.65146E-05 |
| rs6749467 | A | G | -0.01239 | 0.002183 | 0.465814 | 1.40E-08 | 32.22849 | 7.64207E-05 |
| rs77205736 | T | C | 0.013522 | 0.002413 | 0.273909 | 2.10E-08 | 31.39913 | 7.27282E-05 |
| rs77255816 | T | C | 0.031394 | 0.005728 | 0.036507 | 4.20E-08 | 30.04086 | 6.9333E-05 |
| Osteopontin, Arginase-1: | | | | | | | | |
| rs114708313 | T | A | 0.024558 | 0.004478 | 0.06585 | 4.20E-08 | 30.07625 | 7.41973E-05 |
| rs12203592 | T | C | 0.021666 | 0.002591 | 0.212894 | 6.20E-17 | 69.91813 | 0.000157322 |
| rs1372504 | A | G | 0.012291 | 0.002219 | 0.374311 | 3.10E-08 | 30.67374 | 7.07659E-05 |
| rs1537371 | A | C | 0.012371 | 0.002149 | 0.500143 | 8.50E-09 | 33.14877 | 7.65146E-05 |
| rs6749467 | A | G | -0.01239 | 0.002183 | 0.465814 | 1.40E-08 | 32.22849 | 7.64207E-05 |
| rs72642437 | T | C | 0.113396 | 0.019135 | 0.003862 | 3.10E-09 | 35.11871 | 9.89367E-05 |
| rs77205736 | T | C | 0.013522 | 0.002413 | 0.273909 | 2.10E-08 | 31.39913 | 7.27282E-05 |
| rs77255816 | T | C | 0.031394 | 0.005728 | 0.036507 | 4.20E-08 | 30.04086 | 6.9333E-05 |
| Glypican-3: | | | | | | | | |
| rs12203592 | T | C | 0.021666 | 0.002591 | 0.212894 | 6.20E-17 | 69.91813 | 0.000157 |
| rs1537371 | A | C | 0.012371 | 0.002149 | 0.500143 | 8.50E-09 | 33.14877 | 7.65E-05 |
| rs6749467 | A | G | -0.01239 | 0.002183 | 0.465814 | 1.40E-08 | 32.22849 | 7.64E-05 |
| rs77205736 | T | C | 0.013522 | 0.002413 | 0.273909 | 2.10E-08 | 31.39913 | 7.27E-05 |

**Supplementary Table 4**. Genetic variants significantly associated with PM2.5-10 exposure as instrumental variables and biomarkers as outcomes in European population.

| SNP | Effect allele | Other allele | Beta | SE | EAF | *P* | F | R^2^ |
| --- | --- | --- | --- | --- | --- | --- | --- | --- |
| Alpha-fetoprotein: | | | | | | | | |
| rs111308789 | A | T | -0.02194 | 0.004729 | 0.056635 | 3.50E-06 | 21.52391 | 5.14E-05 |
| rs11621531 | A | G | -0.02313 | 0.004487 | 0.063928 | 2.50E-07 | 26.56476 | 6.40E-05 |
| rs116259145 | A | C | 0.030164 | 0.006185 | 0.031996 | 1.10E-06 | 23.78444 | 5.64E-05 |
| rs116816317 | A | G | 0.037649 | 0.007965 | 0.019771 | 2.30E-06 | 22.34258 | 5.49E-05 |
| rs117125329 | G | C | 0.055167 | 0.011992 | 0.008295 | 4.20E-06 | 21.16166 | 5.01E-05 |
| rs117389221 | C | T | -0.04022 | 0.008501 | 0.017253 | 2.20E-06 | 22.38495 | 5.49E-05 |
| rs118101191 | T | G | 0.059791 | 0.011063 | 0.010103 | 6.50E-08 | 29.20813 | 7.15E-05 |
| rs12462492 | T | G | -0.01239 | 0.002496 | 0.257064 | 6.90E-07 | 24.64175 | 5.86E-05 |
| rs13125748 | A | C | 0.011453 | 0.002502 | 0.257878 | 4.70E-06 | 20.95909 | 5.02E-05 |
| rs138141967 | T | G | -0.04294 | 0.009155 | 0.01498 | 2.70E-06 | 21.99721 | 5.44E-05 |
| rs1706918 | A | G | 0.018349 | 0.003677 | 0.098932 | 6.00E-07 | 24.90639 | 6.00E-05 |
| rs17675316 | G | A | 0.036905 | 0.00788 | 0.021631 | 2.80E-06 | 21.93191 | 5.76E-05 |
| rs57048268 | C | A | -0.01102 | 0.002365 | 0.311303 | 3.20E-06 | 21.70542 | 5.21E-05 |
| rs605027 | T | C | -0.01139 | 0.002454 | 0.729529 | 3.40E-06 | 21.56452 | 5.12E-05 |
| rs62079137 | C | T | -0.01735 | 0.003743 | 0.107445 | 3.60E-06 | 21.49039 | 5.77E-05 |
| rs71323440 | T | C | 0.016293 | 0.003408 | 0.114944 | 1.70E-06 | 22.85868 | 5.40E-05 |
| rs76170056 | A | C | 0.016508 | 0.003462 | 0.110936 | 1.90E-06 | 22.73626 | 5.38E-05 |
| rs8006373 | A | T | -0.01758 | 0.003778 | 0.091464 | 3.30E-06 | 21.65915 | 5.14E-05 |
| rs8051340 | G | C | 0.016073 | 0.003326 | 0.122584 | 1.30E-06 | 23.35649 | 5.56E-05 |
| rs9497937 | A | C | -0.01277 | 0.002577 | 0.232437 | 7.20E-07 | 24.55559 | 5.82E-05 |
| rs9997134 | C | T | -0.01047 | 0.002255 | 0.398721 | 3.40E-06 | 21.56995 | 5.26E-05 |
| Osteopontin, Arginase-1: | | | | | | | | |
| rs10152521 | C | T | 0.114394 | 0.023431 | 0.002527 | 1.00E-06 | 23.83512 | 6.60E-05 |
| rs111308789 | A | T | -0.02194 | 0.004729 | 0.056635 | 3.50E-06 | 21.52391 | 5.14E-05 |
| rs1157546 | C | T | -0.0246 | 0.004901 | 0.053047 | 5.20E-07 | 25.20478 | 6.08E-05 |
| rs11621531 | A | G | -0.02313 | 0.004487 | 0.063928 | 2.50E-07 | 26.56476 | 6.40E-05 |
| rs116259145 | A | C | 0.030164 | 0.006185 | 0.031996 | 1.10E-06 | 23.78444 | 5.64E-05 |
| rs116816317 | A | G | 0.037649 | 0.007965 | 0.019771 | 2.30E-06 | 22.34258 | 5.49E-05 |
| rs117125329 | G | C | 0.055167 | 0.011992 | 0.008295 | 4.20E-06 | 21.16166 | 5.01E-05 |
| rs117389221 | C | T | -0.04022 | 0.008501 | 0.017253 | 2.20E-06 | 22.38495 | 5.49E-05 |
| rs118101191 | T | G | 0.059791 | 0.011063 | 0.010103 | 6.50E-08 | 29.20813 | 7.15E-05 |
| rs12462492 | T | G | -0.01239 | 0.002496 | 0.257064 | 6.90E-07 | 24.64175 | 5.86E-05 |
| rs13125748 | A | C | 0.011453 | 0.002502 | 0.257878 | 4.70E-06 | 20.95909 | 5.02E-05 |
| rs138141967 | T | G | -0.04294 | 0.009155 | 0.01498 | 2.70E-06 | 21.99721 | 5.44E-05 |
| rs1706918 | A | G | 0.018349 | 0.003677 | 0.098932 | 6.00E-07 | 24.90639 | 6.00E-05 |
| rs17675316 | G | A | 0.036905 | 0.00788 | 0.021631 | 2.80E-06 | 21.93191 | 5.76E-05 |
| rs57048268 | C | A | -0.01102 | 0.002365 | 0.311303 | 3.20E-06 | 21.70542 | 5.21E-05 |
| rs605027 | T | C | -0.01139 | 0.002454 | 0.729529 | 3.40E-06 | 21.56452 | 5.12E-05 |
| rs62079137 | C | T | -0.01735 | 0.003743 | 0.107445 | 3.60E-06 | 21.49039 | 5.77E-05 |
| rs71323440 | T | C | 0.016293 | 0.003408 | 0.114944 | 1.70E-06 | 22.85868 | 5.40E-05 |
| rs76170056 | A | C | 0.016508 | 0.003462 | 0.110936 | 1.90E-06 | 22.73626 | 5.38E-05 |
| rs8006373 | A | T | -0.01758 | 0.003778 | 0.091464 | 3.30E-06 | 21.65915 | 5.14E-05 |
| rs8051340 | G | C | 0.016073 | 0.003326 | 0.122584 | 1.30E-06 | 23.35649 | 5.56E-05 |
| rs9497937 | A | C | -0.01277 | 0.002577 | 0.232437 | 7.20E-07 | 24.55559 | 5.82E-05 |
| rs9997134 | C | T | -0.01047 | 0.002255 | 0.398721 | 3.40E-06 | 21.56995 | 5.26E-05 |
| Glypican-3: | | | | | | | | |
| rs12462492 | T | G | -0.01239 | 0.002496 | 0.257064 | 6.90E-07 | 24.64175 | 5.86E-05 |
| rs13125748 | A | C | 0.011453 | 0.002502 | 0.257878 | 4.70E-06 | 20.95909 | 5.02E-05 |
| rs605027 | T | C | -0.01139 | 0.002454 | 0.729529 | 3.40E-06 | 21.56452 | 5.12E-05 |
| rs62079137 | C | T | -0.01735 | 0.003743 | 0.107445 | 3.60E-06 | 21.49039 | 5.77E-05 |
| rs71323440 | T | C | 0.016293 | 0.003408 | 0.114944 | 1.70E-06 | 22.85868 | 5.40E-05 |
| rs9497937 | A | C | -0.01277 | 0.002577 | 0.232437 | 7.20E-07 | 24.55559 | 5.82E-05 |

**Supplementary Table 5**. Genetic variants significantly associated with PM10 exposure as instrumental variables and biomarkers as outcomes in European population.

| SNP | Effect allele | Other allele | Beta | SE | EAF | *P* | F | R^2^ |
| --- | --- | --- | --- | --- | --- | --- | --- | --- |
| Alpha-fetoprotein, Osteopontin, Arginase-1: | | | | | | | | |
| rs10498638 | C | T | 0.014014 | 0.002537 | 0.188322 | 3.30E-08 | 30.51287 | 6.00E-05 |
| rs114789974 | A | C | -0.05521 | 0.009635 | 0.010467 | 1.00E-08 | 32.83232 | 6.31E-05 |
| rs13084230 | T | C | -0.01356 | 0.00246 | 0.200319 | 3.50E-08 | 30.39524 | 5.89E-05 |
| rs13122455 | T | C | -0.014 | 0.002464 | 0.199995 | 1.30E-08 | 32.29318 | 6.28E-05 |
| rs140295641 | A | T | -0.03514 | 0.006172 | 0.027357 | 1.30E-08 | 32.40604 | 6.57E-05 |
| rs142169179 | A | G | 0.040185 | 0.00734 | 0.020279 | 4.40E-08 | 29.97094 | 6.42E-05 |
| rs147895162 | C | T | -0.04476 | 0.008107 | 0.015135 | 3.40E-08 | 30.48071 | 5.97E-05 |
| rs182549 | T | C | -0.01242 | 0.002219 | 0.738828 | 2.10E-08 | 31.35735 | 5.96E-05 |
| rs2004679 | C | T | 0.011915 | 0.002138 | 0.307685 | 2.50E-08 | 31.04924 | 6.05E-05 |
| rs2248162 | C | T | 0.011819 | 0.002047 | 0.63988 | 7.80E-09 | 33.33078 | 6.44E-05 |
| rs4788565 | A | G | -0.02192 | 0.003994 | 0.066776 | 4.10E-08 | 30.11083 | 5.99E-05 |
| rs4833095 | C | T | 0.025111 | 0.002406 | 0.206945 | 1.70E-25 | 108.9646 | 0.000207 |
| rs56084453 | G | A | 0.014935 | 0.002412 | 0.209746 | 5.90E-10 | 38.35178 | 7.39E-05 |
| rs60304336 | T | G | 0.027932 | 0.00503 | 0.040906 | 2.80E-08 | 30.84191 | 6.12E-05 |
| rs61620752 | G | T | 0.016069 | 0.002767 | 0.148343 | 6.40E-09 | 33.71969 | 6.52E-05 |
| rs61875074 | C | A | 0.022266 | 0.003841 | 0.073029 | 6.80E-09 | 33.6006 | 6.71E-05 |
| rs6793835 | A | G | -0.01299 | 0.002238 | 0.263796 | 6.60E-09 | 33.66108 | 6.55E-05 |
| rs6867849 | T | A | -0.03148 | 0.005232 | 0.040477 | 1.80E-09 | 36.1985 | 7.70E-05 |
| rs7200852 | A | C | -0.02443 | 0.00445 | 0.054732 | 4.00E-08 | 30.12939 | 6.17E-05 |
| rs74247887 | T | C | 0.037132 | 0.005881 | 0.028721 | 2.70E-10 | 39.86984 | 7.69E-05 |
| rs74805019 | C | G | -0.03071 | 0.005482 | 0.033712 | 2.10E-08 | 31.38607 | 6.15E-05 |
| rs9640029 | T | C | -0.0138 | 0.001974 | 0.478233 | 2.70E-12 | 48.89785 | 9.51E-05 |
| Glypican-3: | | | | | | | | |
| rs13122455 | T | C | -0.014 | 0.002464 | 0.199995 | 1.30E-08 | 32.29318 | 6.28E-05 |
| rs182549 | T | C | -0.01242 | 0.002219 | 0.738828 | 2.10E-08 | 31.35735 | 5.96E-05 |
| rs2004679 | C | T | 0.011915 | 0.002138 | 0.307685 | 2.50E-08 | 31.04924 | 6.05E-05 |
| rs4833095 | C | T | 0.025111 | 0.002406 | 0.206945 | 1.70E-25 | 108.9646 | 0.000207 |
| rs61620752 | G | T | 0.016069 | 0.002767 | 0.148343 | 6.40E-09 | 33.71969 | 6.52E-05 |
| rs6793835 | A | G | -0.01299 | 0.002238 | 0.263796 | 6.60E-09 | 33.66108 | 6.55E-05 |

**Supplementary Table 6**. Genetic variants significantly associated with nitrogen dioxide exposure as instrumental variables and biomarkers as outcomes in European population.

| SNP | Effect allele | Other allele | Beta | SE | EAF | *P* | F | R^2^ |
| --- | --- | --- | --- | --- | --- | --- | --- | --- |
| Alpha-fetoprotein, Osteopontin, Glypican-3, Arginase-1: | | | | | | | | |
| rs10983735 | A | G | 0.016311 | 0.002803 | 0.154461 | 5.90E-09 | 33.86681 | 6.95E-05 |
| rs12203592 | T | C | 0.015941 | 0.002408 | 0.219436 | 3.60E-11 | 43.82627 | 8.71E-05 |
| rs34623735 | T | C | 0.01272 | 0.002157 | 0.33449 | 3.70E-09 | 34.7748 | 7.20E-05 |
| rs7225402 | C | T | -0.02489 | 0.004315 | 0.058308 | 8.00E-09 | 33.27316 | 6.80E-05 |
| rs77205736 | T | C | 0.015406 | 0.002269 | 0.274855 | 1.10E-11 | 46.11078 | 9.46E-05 |

**Supplementary Table 7**. Genetic variants significantly associated with nitrogen oxides exposure as instrumental variables and biomarkers as outcomes in European population.

| SNP | Effect allele | Other allele | Beta | SE | EAF | *P* | F | R^2^ |
| --- | --- | --- | --- | --- | --- | --- | --- | --- |
| Alpha-fetoprotein, Osteopontin: | | | | | | | | |
| rs1217106 | G | A | 0.014557 | 0.002519 | 0.782419 | 7.50E-09 | 33.40472 | 7.22E-05 |
| rs12203592 | T | C | 0.01937 | 0.002457 | 0.219436 | 3.20E-15 | 62.16427 | 0.000129 |
| rs1318845 | C | T | -0.01417 | 0.002596 | 0.200795 | 4.80E-08 | 29.80228 | 6.45E-05 |
| rs6749467 | A | G | -0.01166 | 0.002096 | 0.46467 | 2.60E-08 | 30.95781 | 6.77E-05 |
| rs72808024 | C | A | -0.01703 | 0.002906 | 0.148435 | 4.60E-09 | 34.33572 | 7.33E-05 |
| rs7514956 | C | A | -0.01461 | 0.002652 | 0.186513 | 3.60E-08 | 30.34155 | 6.48E-05 |
| rs77205736 | T | C | 0.013265 | 0.002315 | 0.274855 | 1.00E-08 | 32.84429 | 7.01E-05 |
| rs77255816 | T | C | 0.029905 | 0.005471 | 0.036924 | 4.60E-08 | 29.87975 | 6.36E-05 |
| Glypican-3: | | | | | | | | |
| rs12203592 | T | C | 0.01937 | 0.002457 | 0.219436 | 3.20E-15 | 62.16427 | 0.000129 |
| rs6749467 | A | G | -0.01166 | 0.002096 | 0.46467 | 2.60E-08 | 30.95781 | 6.77E-05 |
| rs72808024 | C | A | -0.01703 | 0.002906 | 0.148435 | 4.60E-09 | 34.33572 | 7.33E-05 |
| rs77205736 | T | C | 0.013265 | 0.002315 | 0.274855 | 1.00E-08 | 32.84429 | 7.01E-05 |
| Arginase-1: | | | | | | | | |
| rs1318845 | C | T | -0.01417 | 0.002596 | 0.200795 | 4.80E-08 | 29.80228 | 6.45E-05 |
| rs6749467 | A | G | -0.01166 | 0.002096 | 0.46467 | 2.60E-08 | 30.95781 | 6.77E-05 |
| rs72808024 | C | A | -0.01703 | 0.002906 | 0.148435 | 4.60E-09 | 34.33572 | 7.33E-05 |
| rs7514956 | C | A | -0.01461 | 0.002652 | 0.186513 | 3.60E-08 | 30.34155 | 6.48E-05 |
| rs77255816 | T | C | 0.029905 | 0.005471 | 0.036924 | 4.60E-08 | 29.87975 | 6.36E-05 |

## Supplementary Figures

**Supplementary Figure 1.** Flow chart of this Mendelian randomization study.


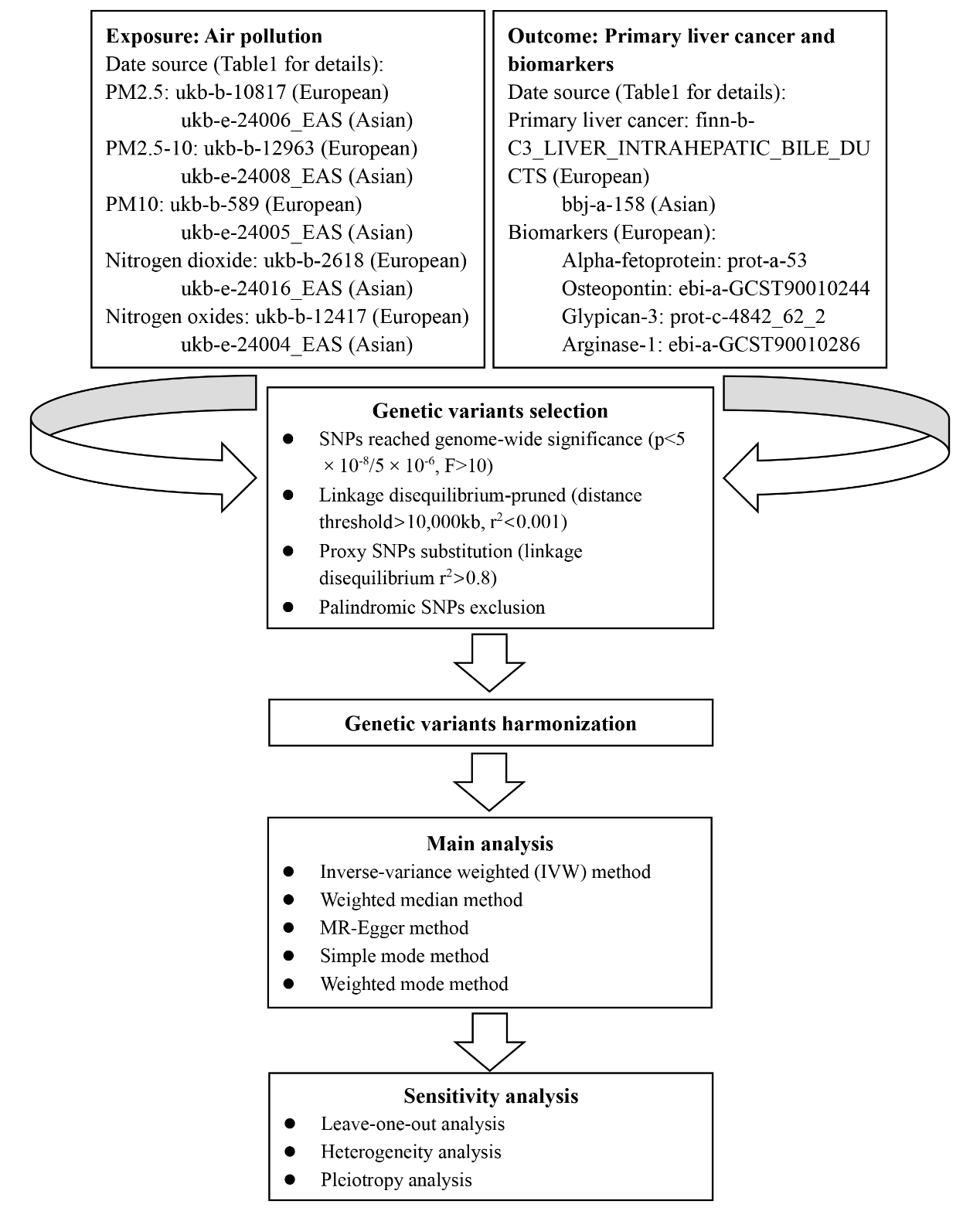


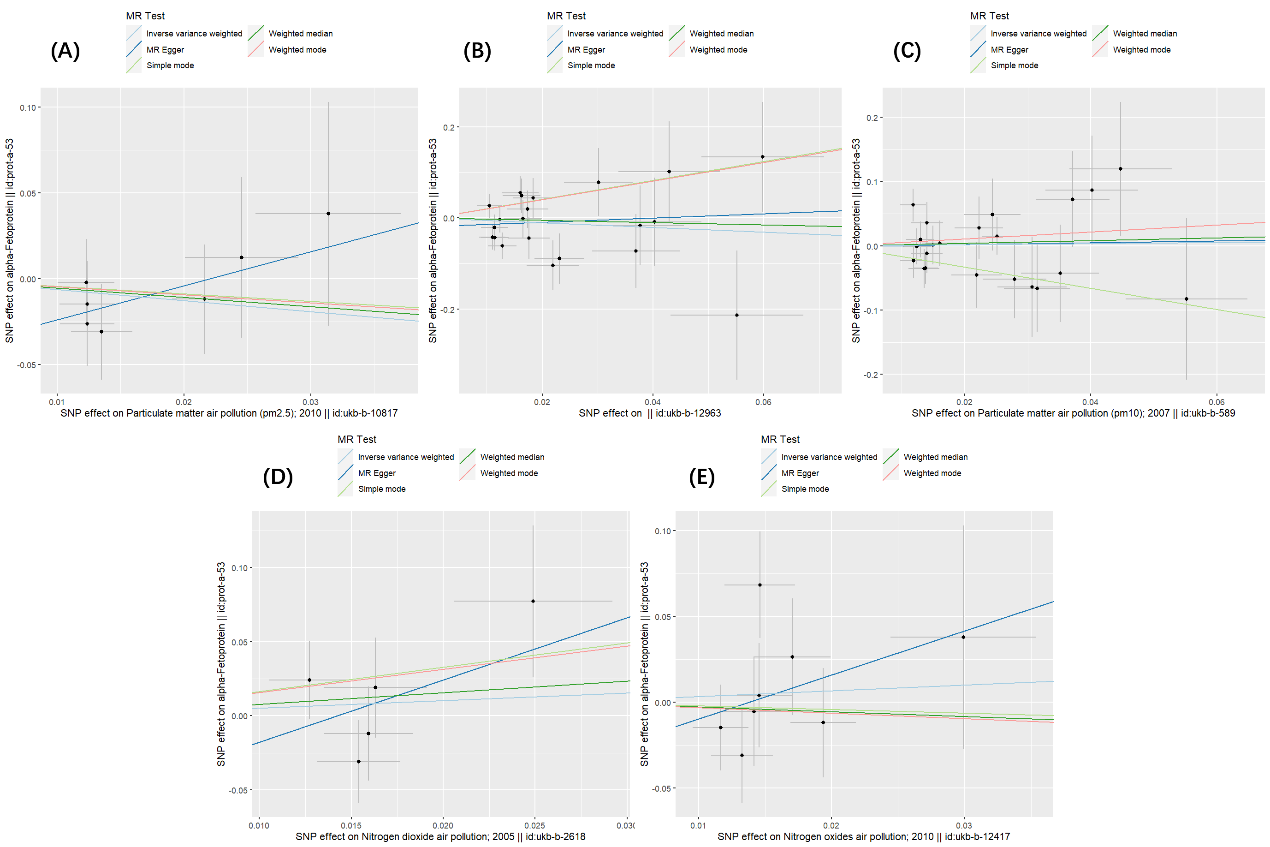


**Supplementary Figure 2**. Scatter plots for causal SNP effect of air pollution (particulate matter, nitrogen dioxide and nitrogen oxides) on Alpha-fetoprotein in European population. (A) PM2.5; (B) PM2.5-10; (C) PM10; (D) Nitrogen dioxide; (E) Nitrogen oxides.


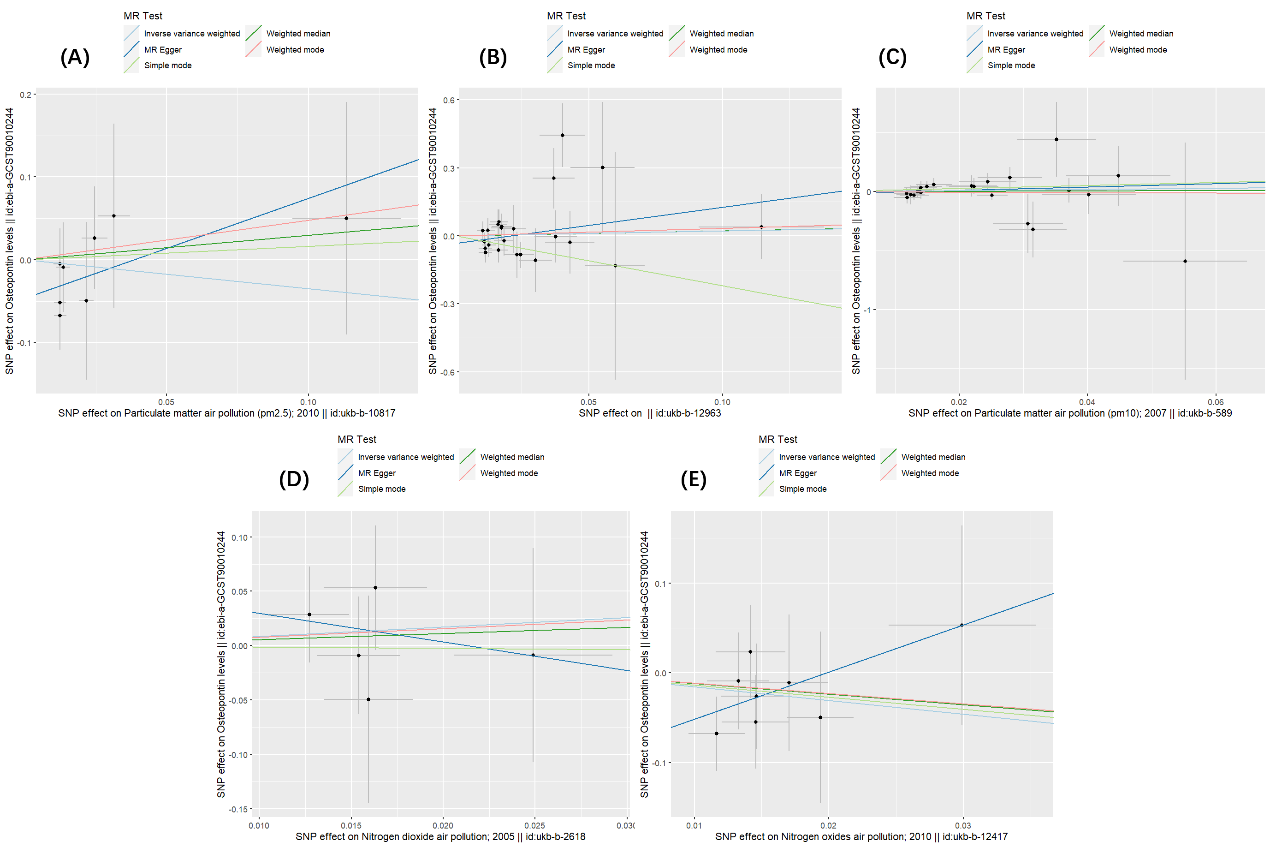


**Supplementary Figure 3**. Scatter plots for causal SNP effect of air pollution (particulate matter, nitrogen dioxide and nitrogen oxides) on Osteopontin in European population. (A) PM2.5; (B) PM2.5-10; (C) PM10; (D) Nitrogen dioxide; (E) Nitrogen oxides.


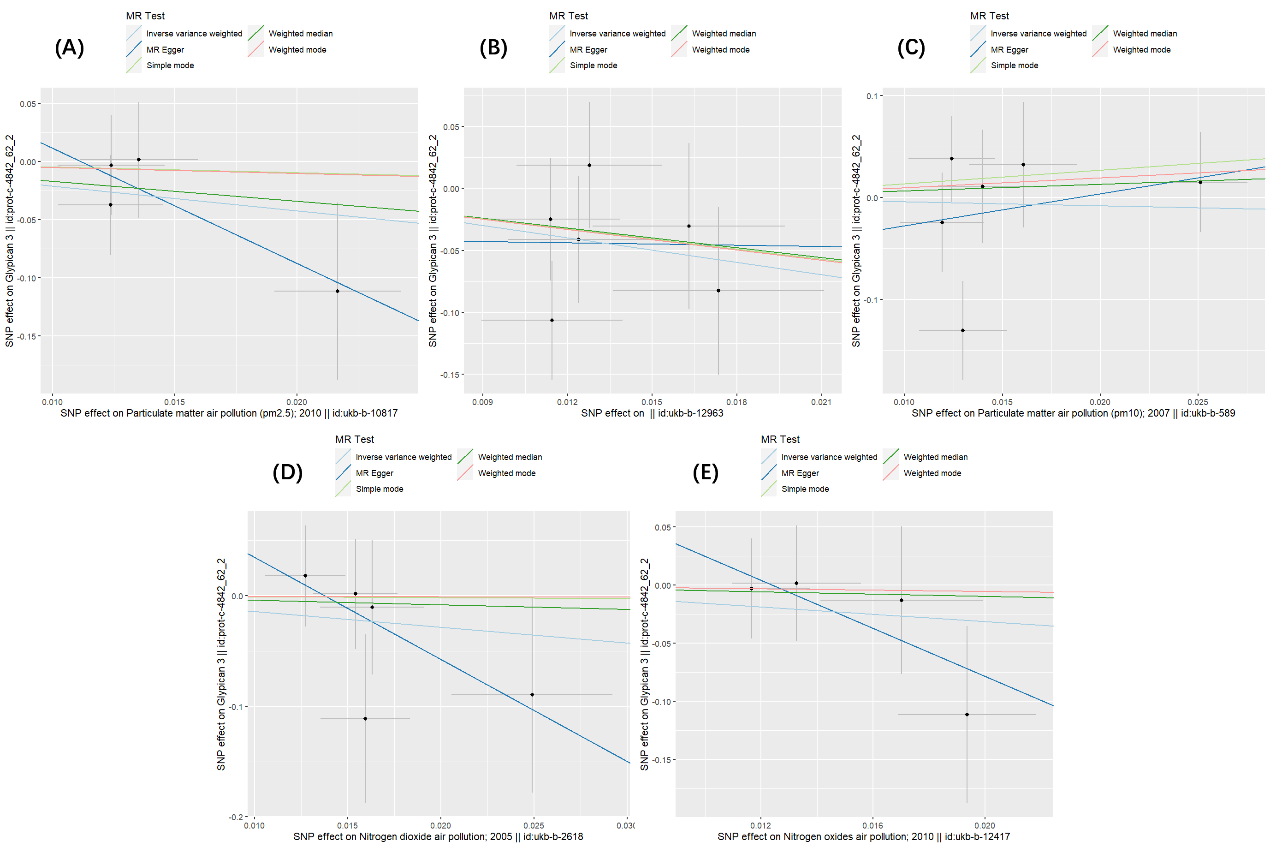


**Supplementary Figure 4**. Scatter plots for causal SNP effect of air pollution (particulate matter, nitrogen dioxide and nitrogen oxides) on Glypican-3 in European population. (A) PM2.5; (B) PM2.5-10; (C) PM10; (D) Nitrogen dioxide; (E) Nitrogen oxides.


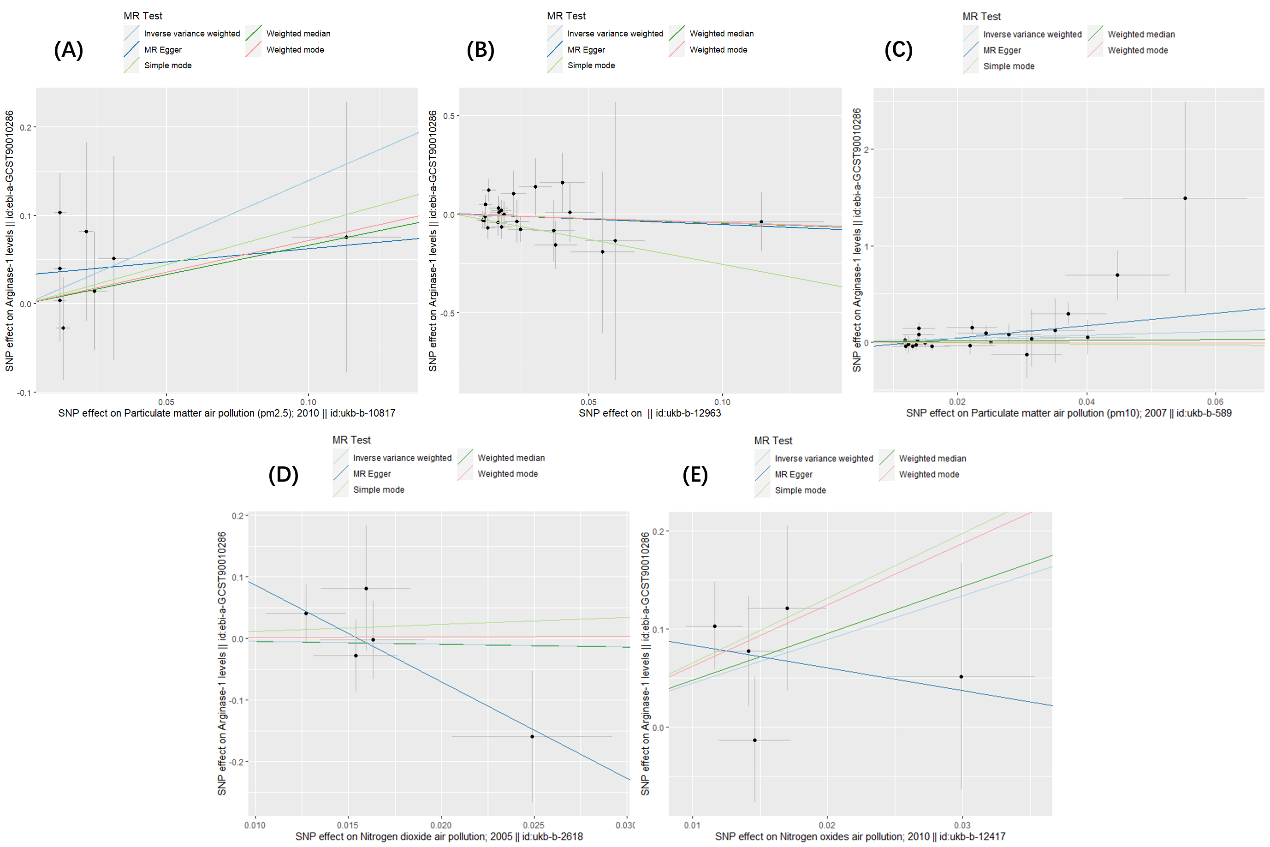


**Supplementary Figure 5**. Scatter plots for causal SNP effect of air pollution (particulate matter, nitrogen dioxide and nitrogen oxides) on Arginase-1 in European population. (A) PM2.5; (B) PM2.5-10; (C) PM10; (D) Nitrogen dioxide; (E) Nitrogen oxides.


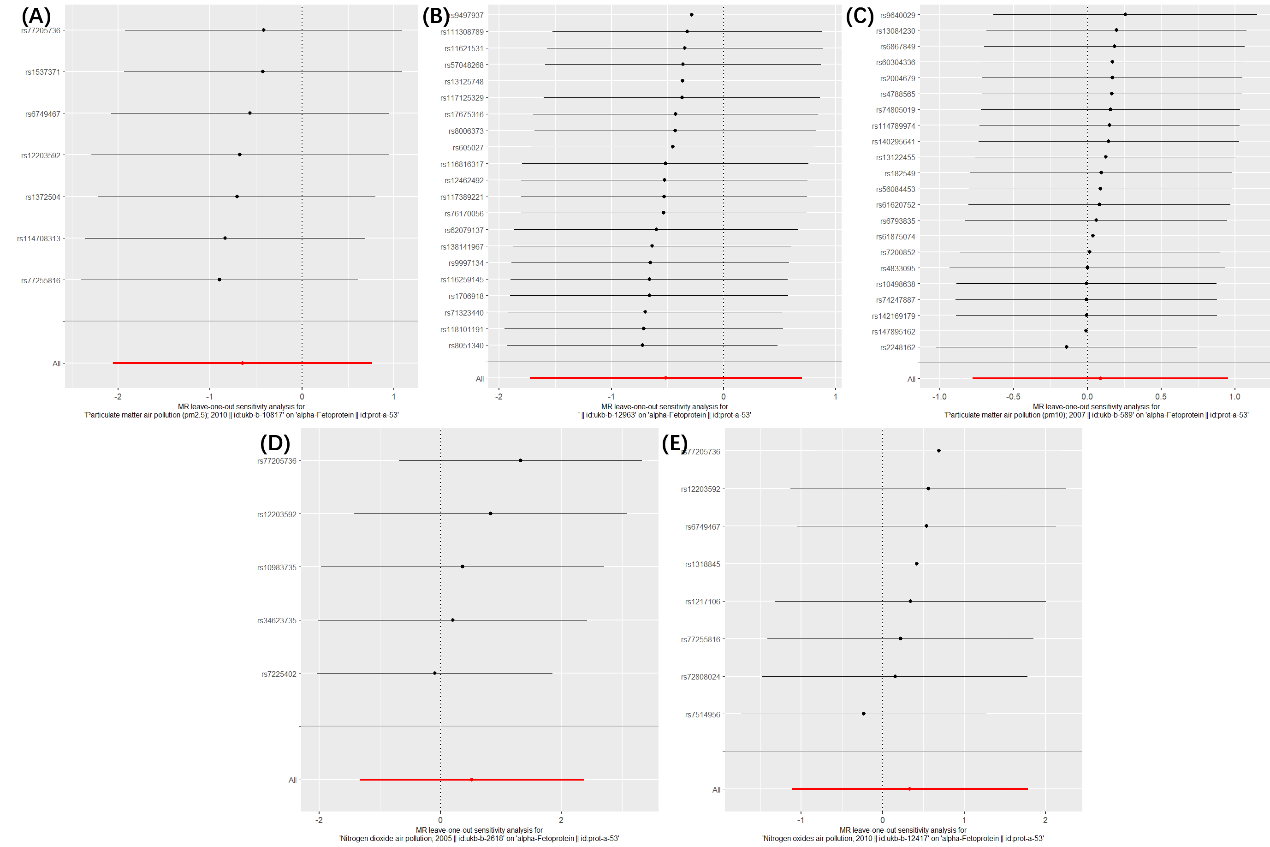


**Supplementary Figure 6**. Forest plots of Leave-one-out analyses for causal SNP effect of air pollution (particulate matter, nitrogen dioxide and nitrogen oxides) on Alpha-fetoprotein in European population. The error bars indicate the 95% confidence interval (CI). (A) PM2.5; (B) PM2.5-10; (C) PM10; (D) Nitrogen dioxide; (E) Nitrogen oxides.


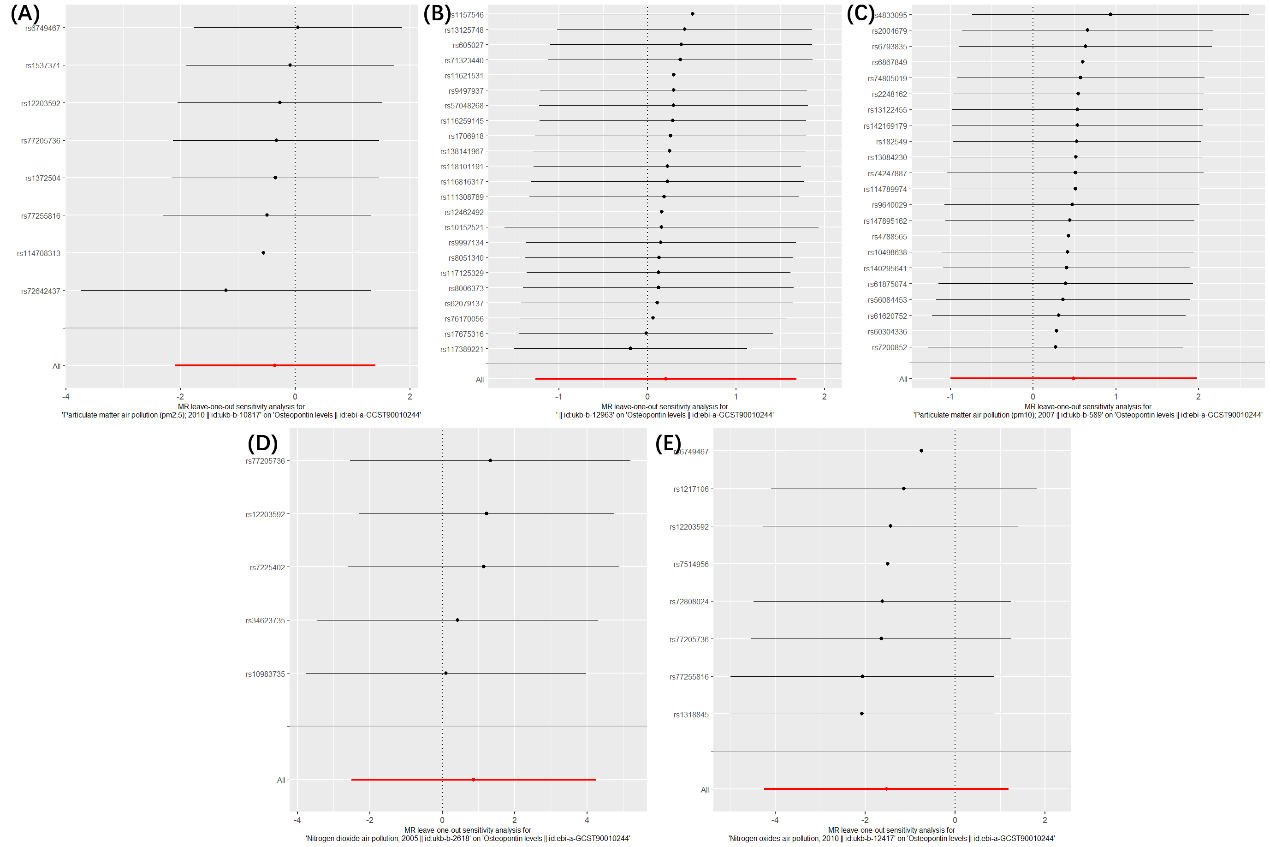


**Supplementary Figure 7**. Forest plots of Leave-one-out analyses for causal SNP effect of air pollution (particulate matter, nitrogen dioxide and nitrogen oxides) on Osteopontin in European population. The error bars indicate the 95% confidence interval (CI). (A) PM2.5; (B) PM2.5-10; (C) PM10; (D) Nitrogen dioxide; (E) Nitrogen oxides.


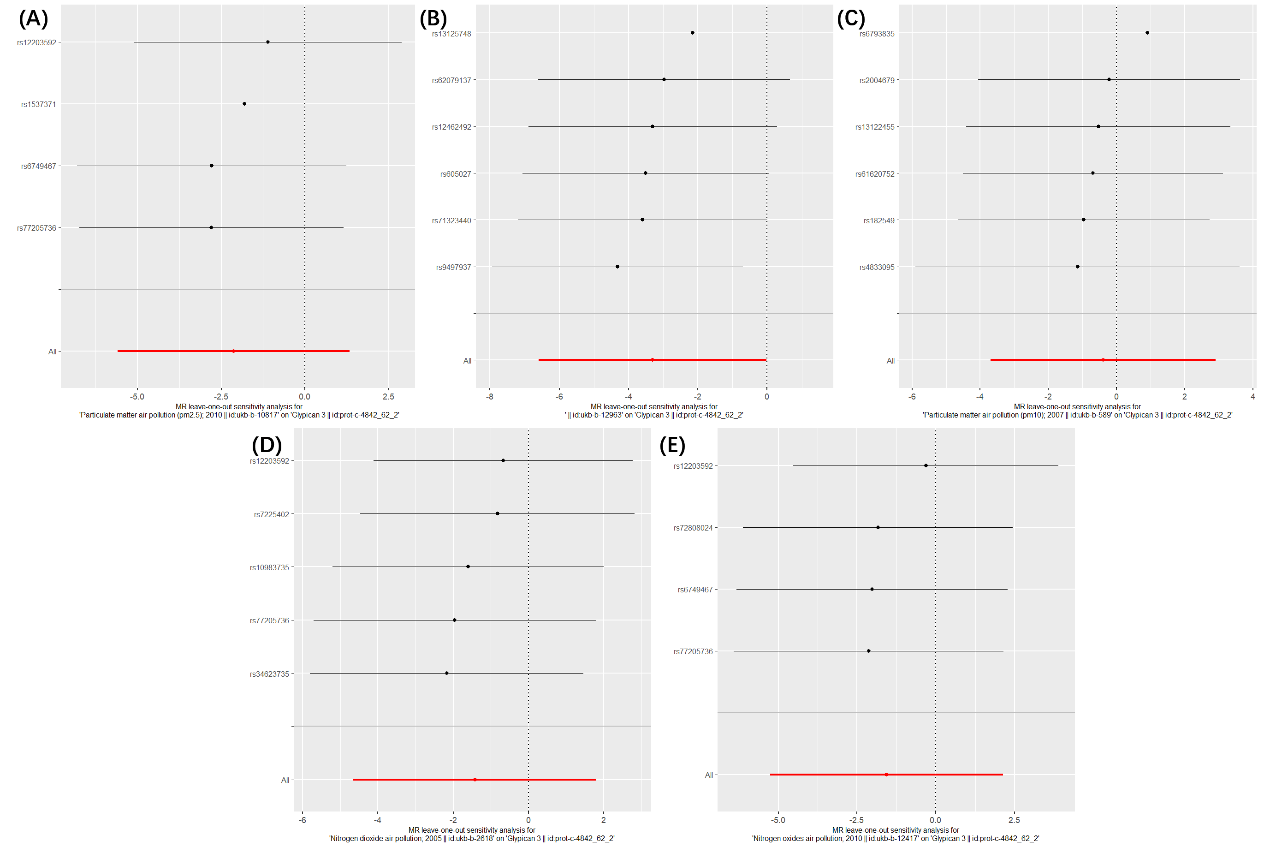


**Supplementary Figure 8**. Forest plots of Leave-one-out analyses for causal SNP effect of air pollution (particulate matter, nitrogen dioxide and nitrogen oxides) on Glypican-3 in European population. The error bars indicate the 95% confidence interval (CI). (A) PM2.5; (B) PM2.5-10; (C) PM10; (D) Nitrogen dioxide; (E) Nitrogen oxides.


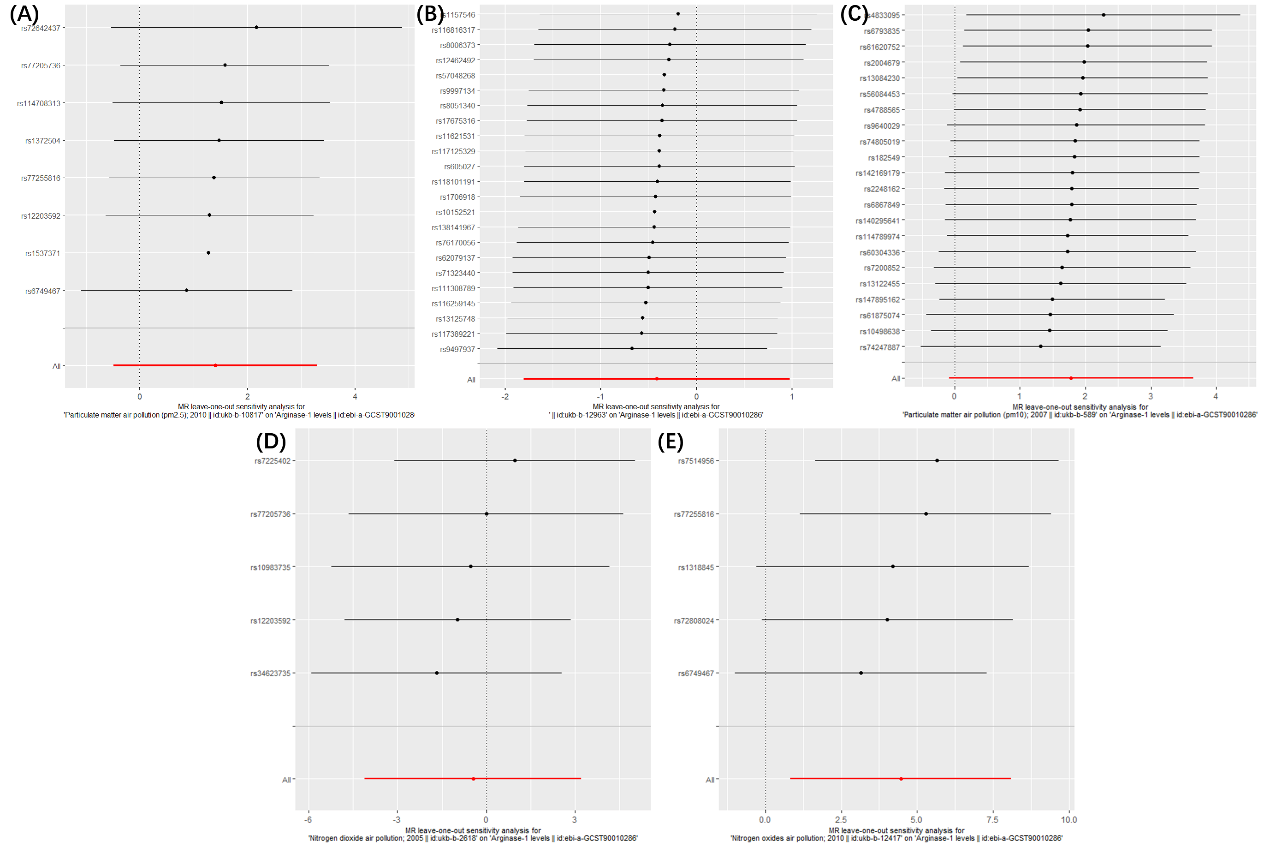
 **Supplementary Figure 9**. Forest plots of Leave-one-out analyses for causal SNP effect of air pollution (particulate matter, nitrogen dioxide and nitrogen oxides) on Arginase-1 in European population. The error bars indicate the 95% confidence interval (CI). (A) PM2.5; (B) PM2.5-10; (C) PM10; (D) Nitrogen dioxide; (E) Nitrogen oxides.
